# Cancer-cell-secreted CXCL11 promoted CD8^+^ T cells infiltration through docetaxel-induced-release of HMGB1 in NSCLC

**DOI:** 10.1186/s40425-019-0511-6

**Published:** 2019-02-11

**Authors:** Qun Gao, Shumin Wang, Xinfeng Chen, Shaoyan Cheng, Zhen Zhang, Feng Li, Lan Huang, Yang Yang, Bin Zhou, Dongli Yue, Dan Wang, Ling Cao, Nomathamsanqa Resegofetse Maimela, Bin Zhang, Jane Yu, Liping Wang, Yi Zhang

**Affiliations:** 1 Biotherapy Center, the First Affiliated Hospital of Zhengzhou University, Zhengzhou, 450052 Henan China; 2 Cancer Center, the First Affiliated Hospital of Zhengzhou University, Zhengzhou, 450052 Henan China; 3 Department of Thoracic Surgery, the First Affiliated Hospital of Zhengzhou University, Zhengzhou, 450052 Henan China; 4 School of Life Sciences, Zhengzhou University, Zhengzhou, Henan 450001 People’s Republic of China; 5 Department of Hematology/Oncology, School of Medicine, Northwestern University, Chicago, USA; 6 Department of Internal Medicine, College of Medicine, University of Cincinnati, Cincinnati, OH 45267 USA; 7 Henan Key Laboratory for Tumor Immunology and Biotherapy, Zhengzhou, 450052 Henan China; 8 Department of Oncology, the First Affiliated Hospital of Zhengzhou University, Zhengzhou, 450052 Henan China

**Keywords:** Docetaxel, CXCL11, CD8^+^ T cells, HER2-CAR T cells; high-mobility group box-1, Non-small cell lung cancer

## Abstract

**Background:**

Chemotherapy combined with immunotherapy becomes the main trend in lung cancer intervention; however, how chemotherapy promotes the immune function remains elusive. Therefore, we sought to determine how chemotherapy promotes the immune function.

**Methods:**

We determined in 100 NSCLC patients the expression of CD8, functional markers (IFN-γ, Granzyme B, and Perforin) and specific chemokines by quantitative real-time reverse transcriptase-PCR. Functional experiments were carried out to check whether docetaxel (DOC), a chemotherapeutic agent, modifies the expression of HMGB1 and CXCL11, and influences the infiltration properties of CD8^+^ T cells to the tumor microenvironment. The mechanism of the release of HMGB1 and CXCL11 was determined by flow cytometry, immunofluorescence and western blotting. In in vivo experiment, we confirmed how DOC enhanced the recruitment of HER2-CAR T cells to tumor sites.

**Results:**

We found that DOC upregulated the expression of chemokine receptor ligand CXCL11 in tumor microenvironment and subsequently enhanced CD8^+^ T cell recruitment. DOC treatment significantly increased HMGB1 release in an ROS-dependent manner. Recombinant protein HMGB1 stimulated the secretion of CXCL11 via NF-κB activation in vitro. Tumors from DOC-treated mice exhibited higher expression of HMGB1 and CXCL11, more HER2-CAR T cell infiltration, and reduced progression, relative to control. Increased HMGB1 and CXCL11 expressions were positively correlated with prolonged overall survival of lung cancer patients.

**Conclusions:**

Our results demonstrate that DOC induces CD8^+^ T cell recruitment to the tumor microenvironment by enhancing the secretion of HMGB1 and CXCL11, thus improving the anti-tumor efficacy, indicating that modulating the HMGB1-CXCL11 axis might be helpful for NSCLC treatment.

**Electronic supplementary material:**

The online version of this article (10.1186/s40425-019-0511-6) contains supplementary material, which is available to authorized users.

## Background

Non-small cell lung cancer (NSCLC) is well known to be sensitive to platinum-based drugs; treatment combinations with taxane family drugs such as DOC has been proven to have clinical benefits [1–3]. DOC exhibits broad antitumor activity by microtubule stabilization, and is currently indicated for the treatment of multiple cancer types [4, 5]. Recently, attention has been paid to the relationship between chemotherapeutic response and tumor immune microenvironment. Our previous studies showed that regulatory T cell subsets significantly decreased after DOC treatment in patients with NSCLC [6], and the percentage of CD39^+^/CD73^+^ myeloid-derived suppressor cells (MDSCs) was decreased with chemotherapy cycles in patients with stable disease or partial response to treatment [7], implying that the therapeutic effect of DOC may involve regulation of immune responses. In addition, Garnett et al. reported that DOC could modulate CD4^+^, CD8^+^, CD19^+^, natural killer cells, and Treg populations in non-tumor-bearing mice, and enhance IFN-γ production by CD8^+^ T cells in a healthy murine model [8]. Collectively, these studies illustrated that DOC is capable of modulating the immune responses.

High numbers of infiltrating cytotoxic T lymphocytes and low numbers of tumor-associated immune suppressor cells correlate with favorable prognosis in some carcinomas [9, 10]. However, the signals controlling the ability of tumor cells to recruit leukocytes are poorly understood. Some anticancer agents, that have mostly been selected based on their therapeutic features to cause tumor cells stress, could thus influence the recruitment of leukocytes, with subsequent reduction in tumor progression [11]. High mobility group box 1 (HMGB1), one damage associated molecular patterns (DAMP), is associated with either anti- or pro-tumor effects depending on the microenvironment and/or model under investigation [11]. As an endogenous factor, HMGB1, derived from dying tumor cells post chemo- or radiation-therapy, has been shown to induce cytokine secretion [12], migration [13], and maturation of dendritic cells to initiate antigen-specific adaptive immune responses [14, 15]. HMGB1 enhanced release of CXCL12 from stromal cells, which subsequently induced robust infiltration of neutrophils and dendritic cells into the tumor, resulting in invasive cancer clearance [16, 17]. On the other hand, as a tumor-promoting agent, tumor cell-released HMGB1 enhanced immunosuppressive cell recruitment, tumor angiogenesis, invasion and metastasis [18]. Especially worth mentioning is that HMGB1 has been reported to play paradoxical roles in promoting immune cell recruitment, even when dependent on the same chemokine. For instance, HMGB1-induced recruitment of TAM depends on CXCL12, which forms a hetero-complex with HMGB1 and acts exclusively through CXCR4 in IKKα WT mice [19, 20]. This directly conflicts with the actions of CXCL12-induced infiltration of neutrophils and dendritic cells, as mentioned previously [16, 17]. Thus, the mechanisms by which HMGB1 modulates the recruitment and functions of infiltrating leukocytes and to what extent this influences the outcome of chemotherapy are still unknown.

In this study, we found that DOC upregulated CXCL11 in the tumor microenvironment and subsequently enhanced the recruitment of CD8^+^ T cells. DOC resulted in a significant HMGB1 release, in an ROS dependent manner. Furthermore, recombinant HMGB1 protein stimulated the secretion of CXCL11 through the activation of NF-κB signaling. This study demonstrates the important role of HMGB1 in the control of CXCL11-induced accumulation of CD8^+^ T cells in NSCLC. Thus, modulating the HMGB1-CXCL11 axis might provide a potential treatment strategy for NSCLC therapy.

## Methods

### Clinical subjects

Fresh tissue samples were collected from 100 patients in the First Affiliated Hospital of Zhengzhou University. Tumor tissues and adjacent normal tissues (at least 3 cm away from the tumor site) were obtained from patients with NSCLC for the isolation of tumor and non-tumor infiltrating leukocytes, RNA extraction, immunohistochemical staining and immunofluorescence detection. The clinicopathologic information, including patient characteristics, tumor size, clinical staging, lymph node involvement, and metastasis is summarized in Additional file 1: Table S1 (*n* = 100). 8 patients received oxaliplatin (120 mg/m^2^) plus docetaxel (60 mg/m^2^ on day 1 and day 8) every three weeks for 1 or 4 cycles. 92 patients were not given this treatment. An additional 6 patients, who have the similar age, same UICC stage, pathologic grade and metastasis state, were also recruited into the study. Detailed patient characteristics are summarized in Additional file 1: Table S2 (*n* = 6). We obtained tissue sections from an additional 9 patients. Detailed patient characteristics are summarized in Additional file 1: Table S3 (*n* = 9). Signed informed consent forms were obtained from all subjects according to the guidelines of the Ethics Committee Board of the First Affiliated Hospital of Zhengzhou University. In all cases the diagnosis of NSCLC carcinoma was confirmed by standard cytopathology or histopathology after biopsy.

### Cell culture and reagents

Cell lines (i.e., NCI-H460, A549) were purchased from American type culture collection (ATCC, USA). Cell lines were grown and maintained using RPMI-1640 (Hyclone, USA) supplemented with 10% heat inactivated-FBS (GIBCO, Auckland, NZ) in an incubation system at 37 °C with 5% CO2. DOC was obtained from Shanghai Sunve Pharmaceutical Co. Ltd. L-OHP was obtained from Shenzhen Sea King biological engineering Limited by Share Ltd. Recombinant protein HMGB1was purchased from BioLegend (San Diego, CA, USA).

### Isolation of tumor-infiltrating lymphocytes (TILs) and peripheral blood lymphocytes (PBLs)

RPMI-1640 (Invitrogen Life Technologies, Carlsbad, CA, USA) was used to wash freshly sampled tumor tissues 2 times to remove traces of blood. Tissues were cut into small pieces and incubated with 300 μg/ml collagenase (Roche, Indianapolis, IN, USA) and 50 μg/ml deoxyribonuclease I (Sigma-Aldrich, St Louis, MO, USA) for 2 h at 37 °C. Following this, samples were mechanically disaggregated, supernatants were collected and TILs were separated by centrifugation at 2500 rpm for 25 min on Ficoll-Paque Plus (Sigma-Aldrich, Saint Quentin- Fallavier, France). PBLs were isolated from heparinized blood samples by Ficoll-Paque Plus density centrifugation as described [21].

### Flow cytometry

TILs and PBLs were suspended in flow buffer (PBS containing 2% fetal bovine serum), and incubated with anti-CD3 and anti-CD8 (Biolegend, San Diego, CA, USA) against surface antigens for 30 min at 4 °C in the dark, with a subsequent wash in flow buffer, spin and resuspension. Cells were then fixed, permeabilized and stained with anti-IFN-γ, anti-Perforin and anti-Granzyme B antibody (Biolegend, San Diego, CA, USA) for 30 min at 4 °C in the dark at each step. After staining, cells were analyzed using a BD CantoII flow cytometer (Becton Dickinson, San Jose, CA, USA). To detect nonspecific signals, concentration- and isotype-matched nonspecific antibodies were used.

### Quantitative real time polymerase chain reaction (qRT-PCR)

Total RNA was extracted from small pieces of tumor or marginal tissues using Trizol solution (Invitrogen, Carlsbad, CA, USA). RNA from each sample was reverse-transcribed using Prime Script RT reagent Kit (Takara Bio, Otsu, Shiga, Japan). Subsequently, the expression levels of target genes were assessed. Primers are listed in Additional file 1: Table S5. SYBR Premix Ex Taq II (TaKaRa, Japan) based real-time PCR was performed with an Agilent Mx3005P. The delta Ct method was used to calculate relative expression levels. The house-keeping gene GAPDH was used as reference.

### Immunohistochemistry

Formalin-fixed, paraffin-embedded sections (3 μm) were baked at 60 °C for 1 h, deparaffinized in xylene, then rehydrated through graded alcohol and washed briefly in tap water. Endogenous peroxidase was blocked by incubating in methanol containing 0.3% hydrogen peroxide for 30 min. To retrieve antigenicity, sections were boiled in 10 mmol /L citrate buffer (pH 5.8) for 30 min in microwave (800 W). Following that, sections were incubated with goat serum diluted in PBS (pH 7.4) for 30 min at room temperature. Subsequently, sections were incubated at 4 °C over- night with the primary antibodies specific for HMGB1 (dilution 1:400) or CXCL11 (dilution 1:500) (Abcam, Cambridge, MA, USA). Sections were rinsed with fresh PBS and incubated with horseradish peroxidase-linked secondary antibodies at room temperature for 30 min. Finally, sections were stained with DAB substrate (Dako, Carpinteria, CA, USA) and counterstained with Mayer’s hematoxylin. Photos were recorded under microscopy (Leica, Wetzlar, Germany).

### HMGB1 silencing

Two sequences of short interference RNA (SiRNA) to silence HMGB1 gene were purchased from GenePharma, Shanghai, China. The following sequences were chosen: SiHMGB1–1. 5’-GCAGCCCUAUGAAAGAAATT-3′; and SiHMGB1–2.5’-GCUGAAAAGAGCAAGAA AATT-3′; SiScr. 5’-UUCUCCGAACGUGUCACGUTT-3’was used as a control. The SiHMGB1 was transfected into A549 (ATCC, USA) cells following standard protocol using Lipofectamine 2000 (Invitrogen, Carlsbad, CA, USA).

### Western blotting

Protein extractions from Control- or rHMGB1-treated tumor cells were analyzed. Cells were washed with ice-cold phosphate-buffered saline and harvested in a RIPA lysis buffer pulsed with phenylmethanesulfonyl fluoride (PMSF). For NF-κB signaling pathway in regulating CXCL11 production, the sorted tumor cells were directly subjected to protein extraction (basal state). Primary antibodies include p-NF-κB (Cell Signaling, 1:1000 dilution) and β-actin (Sigma, 1:4000 dilution). Anti-mouse/rabbit secondary antibodies were purchased from Cell Signaling (1:4000 dilution).

### Purification of T lymphocytes

TILs were isolated from fresh tumor tissues of NSCLC patients as the protocols described above. T lymphocytes were suspended in flow buffer, stained with anti-CD8 (Biolegend, San Diego, CA, USA) for 20 min at 4 °C in dark and rinsed. CD8^+^ T lymphocytes were independently isolated using MoFlo XDP cytometer (Beckman Coulter Inc., Indianapolis, IN, USA).

### Leukocyte chemotaxis

Directional migration of CD8^+^ T lymphocytes isolated from TILs was evaluated in Costar Transwell permeable polycarbonate supports (5 μm pores) in 24-well plates (Corning Inc., Corning, NY, USA). Supernatant from cultured tumor tissue was used as a positive control, RPMI-1640 media alone as a negative control. Boyden chamber assays were performed as described using CD8^+^ T cells from lung cancer patients [21]. When indicated, mAbs against CXCL11 (Abcam, Cambridge, MA, USA. 100 ng/ml), recombinant CXCL11 (BioLegend, San Diego, CA, USA. 1 μg/ml), or mAbs against CXCR3 (Abcam, Cambridge, MA, USA. 100 ng/ml) were added. The plates were incubated at 37 °C in a 5% CO_2_ atmosphere for 4 h. After that, cells in the bottom chambers were collected and the number of CD8^+^ T cells was calculated by flow cytometry.

### Elisa

Cells were seeded in 24-well plates at a density of 2 × 10^5^ cells/well. Cells were treated with DOC for 12 or 24 h. CXCL11 secretion was analyzed in 100 μl of conditioned medium according to manufacturer protocol (R&D Systems) [22].

### ROS measurements by flow cytometry

Cells were pretreated with DOC and different inhibitors. Pre-treated cells were washed with 1x PBS, and harvested by trypsinization. Cells were then treated with c-H2DCFDA (5 μM, ThermoFisher Scientific) for 30 min at 37 °C to assess hydrogen peroxide (H2O2)-mediated oxidation of the fluorescent compound DCF. Fluorescence of oxidized DCF was measured using flow cytometry (BD FACS Calibur) at excitation wavelength of 480 nm and emission wavelength of 525 nm [23].

### In situ immunofluorescence (IF) staining of TILs

The lung tissue samples were formalin-fixed and paraffin-embedded sections were obtained. Before incubation with antibodies, slides were pretreated by dewaxing with xylene and ethanol. Staining for CD8 (dilution 1:500) and CXCR3 (dilution 1:200) (Abcam, Cambridge, MA, USA) was performing using non-labeled primary antibodies followed by fluorophore-labeled secondary antibodies (dilution 1:1000) (Abcam, Cambridge, MA, USA). In each case, we checked that secondary antibodies did not cross-react with unrelated primary antibodies used in the combination. Then the slides were counterstained with 4′, 6-diamidino-2-phenylindole dihydrochloride (DAPI; Roche Diagnostic, Mannheim, Germany) in mounting medium for 10 min.

### Generation of gene-modified CAR-T cells by retroviral transduction and functional assays

The viral packaging Ampho cells containing empty (control) pMIG II or pMIG II-anti- HER2-CAR retroviral vector was generated as described previously [24]. The CAR construct used in this study was a second generation CAR comprised of an extracellular single chain variable fragment (scFv) specific for human-HER2, a CD8 hinge region, trans-membrane and intracellular CD28 and CD3ζ domains. Then the activated CD8^+^ T cells were retrovirally transduced with retroviral vectors either with HER2-CAR or with control construct [25]. After transduction, T cells were maintained in supplemented RPMI media with IL-2 (100 U/mL). For in-vitro co-culture assays to determine antigen-specific cytokine secretion by the transduced T cells, FSC was performed to determine the amount of IFN-γ secreted following 24 h co-culture of the transduced T cells with tumor cells A549 [26].

### Nude mouse xenograft assay

Female BALB/c nude mice (5 weeks) were raised in the animal facility of the Experimental Animal Center, Zhengzhou University, under sterile conditions in air-filtered containers. 2 × 10^6^ A549 cells in 100 mL of PBS were injected subcutaneously into nude mice. The nude mice developed tumor nodules approximately 9 days later; they were then randomly divided into 4 groups: untreated, DOC treated, HER2-CAR T cell treated, or DOC in combination with HER2-CAR T cell treated groups. The PBS-treated group served as the negative control. DOC was injected intraperitoneally on day 10 at a dose of 10 mg/kg. HER2-CAR T cells (6 × 10^6^) were injected intravenously on day 11. After 3 days, one part of animals was sacrificed and the tumors were excised and analyzed by immunohistochemistry and qRT-PCR. The other part of animals was used to analyze the tumor volume [27].

### Statistical analysis

Analyses were performed using SPSS Statistics Program. Data were expressed as mean ± SEM. Student’s t-test and one-way ANOVA were conducted to compare the differences between variables, as appropriate. If the distribution of data was not normal, the Mann-Whitney rank-sum test was employed for the nonparametric analysis. Spearman’s test was conducted to determine the correlation between chemokine genes and T cell-associated markers. The expressions of HMGB1 and CXCL11 were evaluated based on the intensity (0 = none, low = 1, moderate = 2 and high = 3) of protein staining and the density (0% = 0, 1–40% = 1, 41–75% = 2 and > 76% = 3) of positive cells. The total scores of sections were calculated by multiplying intensity and density. The samples that scored 5 or over were regarded as strong expression. A χ2-test was used for correlation analysis between HMGB1 and CXCL11 expression of patients with NSCLC. According to the median ranking of genes or protein levels, the patients were divided into low expression and high expression groups. Kaplan–Meier survival curves and log-rank test survival analysis was used to evaluate overall survival. Values with *P* < 0.05 were considered significant.

## Results

### Higher number and function of infiltrating CD8^+^ T cells in tumor tissue of NSCLC was related to better survival

**Fig. 1:**
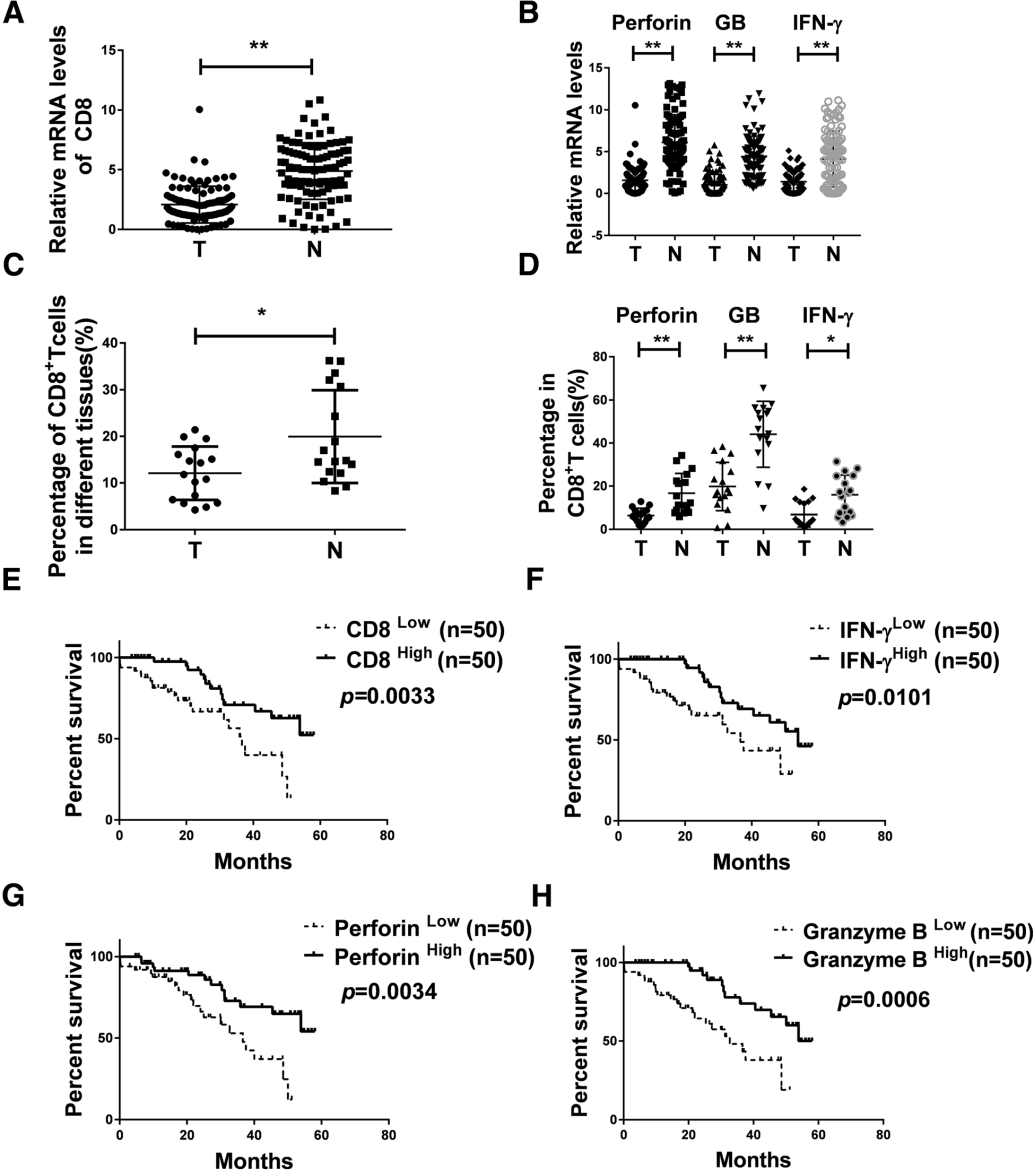
Reduced number and function of infiltrating CD8^+^ T cells in tumor tissues of NSCLC than in the adjacent normal tissues. **a**, **b** The expression of CD8, Perforin, Granzyme B and IFN-γ was detected in samples from NSCLC patients (*n* = 100) using qRT-PCR. Data are given as means ± SEM. Significance is noted as **p < 0.0001. **c**, **d** Percentage of CD8, Perforin, Granzyme B and IFN-γ in samples from NSCLC patients via flow cytometry (*n* = 17), Data are given as means ± SEM. Significance is noted as *p < 0.05; **p < 0.0001. **e**, **f**, **g**, **h** Kaplan–Meier analysis of CD8, IFN-γ, Granzyme B and Perforin expression in NSCLC patients. n(total) = 100

To explore the number and function of infiltrating CD8^+^ T cells in tumor tissue of NSCLC, we detected the relative expression levels of CD8 and functional markers by qRT-PCR assay in NSCLC tissue specimens and adjacent normal tissues from 100 patients. As shown in Fig. 1a and b, the CD8^+^ T cell and the functional markers IFN-γ, Granzyme B, and Perforin were lower in lung cancer tumor sites compared to adjacent normal tissues [CD8 *P* < 0.0001, IFN-γ *P* < 0.0001, Granzyme B *P* < 0.0001, Perforin P < 0.0001]. The results were further validated at the protein level as detected by flow cytometry. The percentage of CD8, IFN-γ, Granzyme B and Perforin were 12.0, 6.4, 19.8 and 6.9% in tumor tissues respectively, compared to 19.9, 16.7, 44.1 and 16% in adjacent normal tissues (*n* = 17) (Fig. 1c, d and Additional file 1: Figure S1A, B). As shown in Fig. 1e, overall survival was positively correlated with the amount of infiltrating CD8^+^ T cells (*n* = 100). Furthermore, there were significant differences in overall survival in term of the levels of IFN-γ, Perforin and Granzyme B as well (*n* = 100) (Fig. 1f-h). Thus, upregulation of CD8 and its functional indicators IFN-γ, Perforin and Granzyme B may be some of the phenotypes of NSCLC with better survival.

### CD8^+^ T cells were recruited by upregulated CXCL11 with DOC-based chemotherapy in tumor of NSCLC patients

**Fig. 2:**
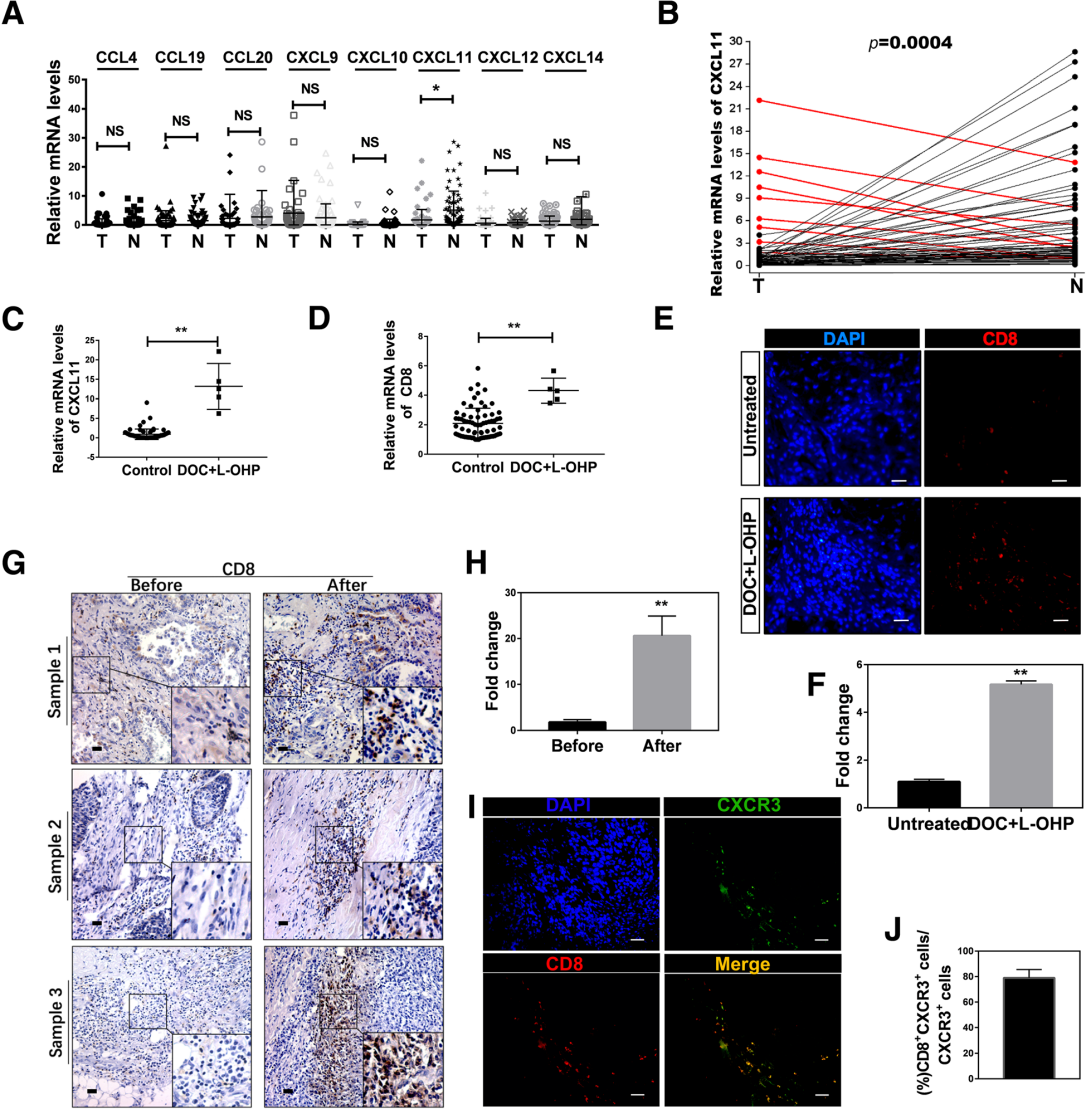
CXCL11 was highly expressed in NSCLC patients with DOC-based chemotherapy, and promoted the accumulation of CD8^+^ T cells. **a** Expression levels of chemokine expression in tumor tissue (T) and adjacent normal tissues (N). Chemokine gene expression was validated in tumor tissues (T) and adjacent normal tissues (N) using qRT-PCR analysis. Statistical differences between groups were determined by the Mann-Whitney rank-sum test (*n* = 77). *P < 0.05; NS, non-significant (*P* > 0.05). **b** Expression of CXCL11 mRNA in lung cancer tumor tissues and adjacent tissue, as detected by qRT-PCR. Samples with CXCL11 in higher in tumor tissue than adjacent tissue are highlighted in red. **c**, **d** The comparative expression of CXCL11 and CD8 in non DOC combined with L-OHP group (*n* = 72) and DOC combined with L-OHP group (*n* = 5). Data are given as means ± SEM. Significance is noted as **p < 0.0001. **e**, **f** Immunofluorescence staining using anti-CD8 mAbs in sections from untreated (without DOC+L-OHP treatment) group and chemotherapy (DOC combined with L-OHP) group patients, and the differences were significant (*n* = 5). Scale bars, 50 μm. **g**, **h** Immunohistochemistry staining of CD8 in sections from patient samples before chemotherapy and after chemotherapy (DOC combined with L-OHP), and the differences were significant (*n* = 9). Scale bars, 100 μm. **i**, **j** Immunofluorescence staining of CD8 (green), CXCR3 (red) and DAPI (blue) in lung tumor tissue (*n* = 5). Scale bars, 50 μm

Chemokines in the tumor microenvironment are considered to play a key role in recruiting immune cells to tumor sites [28]. We analyzed the tumors and adjacent normal tissues for the presence of specific chemokines, and found that CXCL11 was mostly significantly decreased in tumor tissues (Fig. 2a, *n* = 77). We therefore proposed that CXCL11 might be able to reset the tumor microenvironment by modulating CD8^+^ T cell accumulation. We performed a comparative analysis of CXCL11 in tumor versus adjacent normal tissue from patients with NSCLC, and identified nine patients with increased CXCL11 expression in tumor tissues compared to adjacent normal tissue samples (Fig. 2b). After carefully looking into the patients’ records, we found that five of the nine patients experienced 1 or 4 cycles of DOC-based chemotherapy [DOC combined with oxaliplatin (L-OHP)] prior to operation, one patient had neoadjuvant radiotherapy before the operation, and the other three cases had no preoperative treatment (Additional file 1: Table S4, *n* = 9). We compared the expression of CXCL11 between the chemotherapy group (DOC combined with L-OHP) (*n* = 5) and the non DOC combined with L-OHP treatment group (*n* = 72), and found that DOC combined with L-OHP treatment increased the intratumoural CXCL11 approximately 10-fold in the chemotherapy group (0.9224 ± 0.1593 vs 13.19 ± 2.627) (Fig. 2c). Further analysis showed that the infiltration of CD8^+^ T cells in the DOC combined with L-OHP treatment group was much higher in lung cancer tumor sites (Fig. 2d). In order to further verify that DOC combined with L-OHP treatment could enhance the levels of CXCL11 and CD8, we found additional 6 patients who have the similar age, same UICC stage, pathologic grade and metastasis state. 3 of the 6 patients were treated with DOC combined with L-OHP for 4 cycles and the other 3 did not have any treatment before the operation. The levels of CXCL11 and CD8 were detected and the results showed that the group treated with DOC combined with L-OHP had higher levels of CXCL11 and CD8 (Additional file 1: Figure S1C, D). The immunofluorescence assay of CD8 in tumor tissues showed similar results (Fig. 2e, f). Detailed patient characteristics were summarized in Additional file 1: Table S2 (*n* = 6). The preoperative diagnostic puncture specimens (before DOC-based chemotherapy) and postoperative specimens after chemotherapy (after DOC-based chemotherapy) were used to detect the expression of CD8 by IHC. The results showed that the infiltration of CD8^+^T cells significantly increased in the postoperative specimens after DOC-based chemotherapy (Fig. 2g, h). Detailed patient characteristics were summarized in Additional file 1: Table S3 (*n* = 9).

We showed that CD8 and CXCL11 have a positive correlation based on the TCGA data (r = 0.6655, Additional file 1: Figure S1E). CXCR3, the ligand of CXCL11, exhibits potential antitumor activity by attracting cytotoxic T lymphocytes [29, 30]. The co-localization of CD8 and CXCR3 in lung tumor tissues was observed (Fig. 2i, j). The role of CXCL11 in T lymphocyte chemotaxis was assessed by migration assay using transwell plates. As shown in Additional file 1: Figure S1F, G, rCXCL11 and supernatant from cultured tumor tissues derived from chemotherapy-treated patients enhanced the migrating ability of the CD8^+^ T lymphocytes. Blocking with neutralizing antibodies against CXCL11 and CXCR3 reduced the migrating ability of the CD8^+^ T lymphocytes. These observations support the hypothesis that the CXCL11-CXCR3 axis is critical for the migration of CD8^+^ T lymphocytes. Collectively, the chemokine CXCL11 is highly expressed in chemotherapy patients treated with DOC combined with L-OHP, and can promote recruitment of CD8^+^ T lymphocytes.

### CXCL11 was induced by DOC, but not L-OHP

**Fig. 3:**
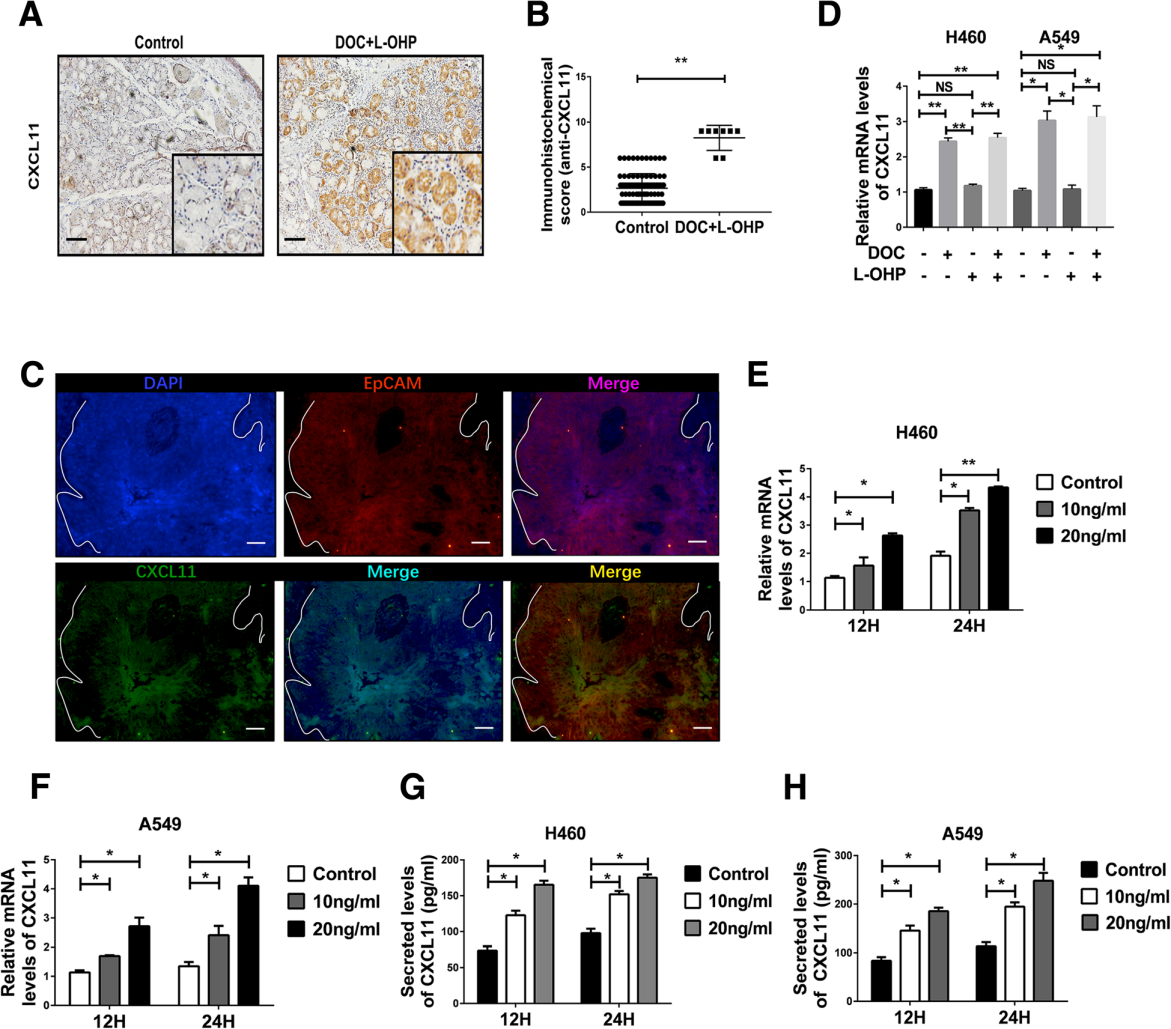
CXCL11 was induced from tumor cells by DOC, but not L-OHP. **a** Representative immunostainings using anti-CXCL11mAbs in sections from untreated (*n* = 92) and chemotherapy group (*n* = 8) patients. Figure panel pairs represents images taken with different zooming options; scale bars, 100 μm. **b** Statistical analysis of the expression of CXCL11 in tumor tissues detected by IHC. n(total) = 100. **p < 0.0001. **c** Immunofluorescence staining of EpCAM and CXCL11 show that CXCL11 is mainly expressed in tumor cells (*n* = 5). Scale bars, 50 μm. (D) CXCL11 levels in lung cancer cells in control group vs DOC (10 ng/ml) or/and L-OHP (20 ng/ml) treated group assessed by RT-PCR. Docetaxel (DOC), but not oxaliplatin (L-OHP), induces upregulation of CXCL11 in vitro. Statistical differences between groups were determined by the Student t test. *p < 0.05; **p < 0.0001; NS, non-significant(*P* > 0.05). **e**, **f**, **g**, **h** Dose and time effects of DOC on RNA and protein expression of CXCL11 in A549 and H460 lung cancer-derived cells. Data are given as means ± SEM. Significant differences between each group are indicated by **p < 0.0001 or *p < 0.05. Experiments were independently repeated three times

Immunohistochemistry was performed to verify that there was a higher expression of CXCL11 protein in lung cancer tumor sites from patients treated with DOC combined with L-OHP, compared to tumor sites derived from patients who did not undergo chemotherapy (Fig. 3a, b). Interestingly, co-localization of immunofluorescence and immunohistochemistry of serial sections indicted that CXCL11 was mostly located within the tumor cells (Fig. 3c and Additional file 1: Figure S1H). Next, we checked the expression of CXCL11 in tumor cells after treatment with DOC or L-OHP. Only DOC induced the expression of CXCL11; L-OHP did not induce expression of CXCL11 (Fig. 3d). Similar results were obtained in two different cell lines A549 and NCI-H460 (Fig. 3d). CXCL11 protein levels were dependent on dose course of DOC treatment (Fig. 3e, f). Similar results were obtained using ELISA assays (Fig. 3g, h). However, DOC didn’t influence the levels of CXCL10 and CXCL9 in tumor cells (Additional file 1: Figure S2A-D). Collectively, these data suggest that the secretion of CXCL11 by tumor cells is induced by DOC rather than L-OHP.

### DOC induced HMGB1 release through a positive feedback process

**Fig. 4:**
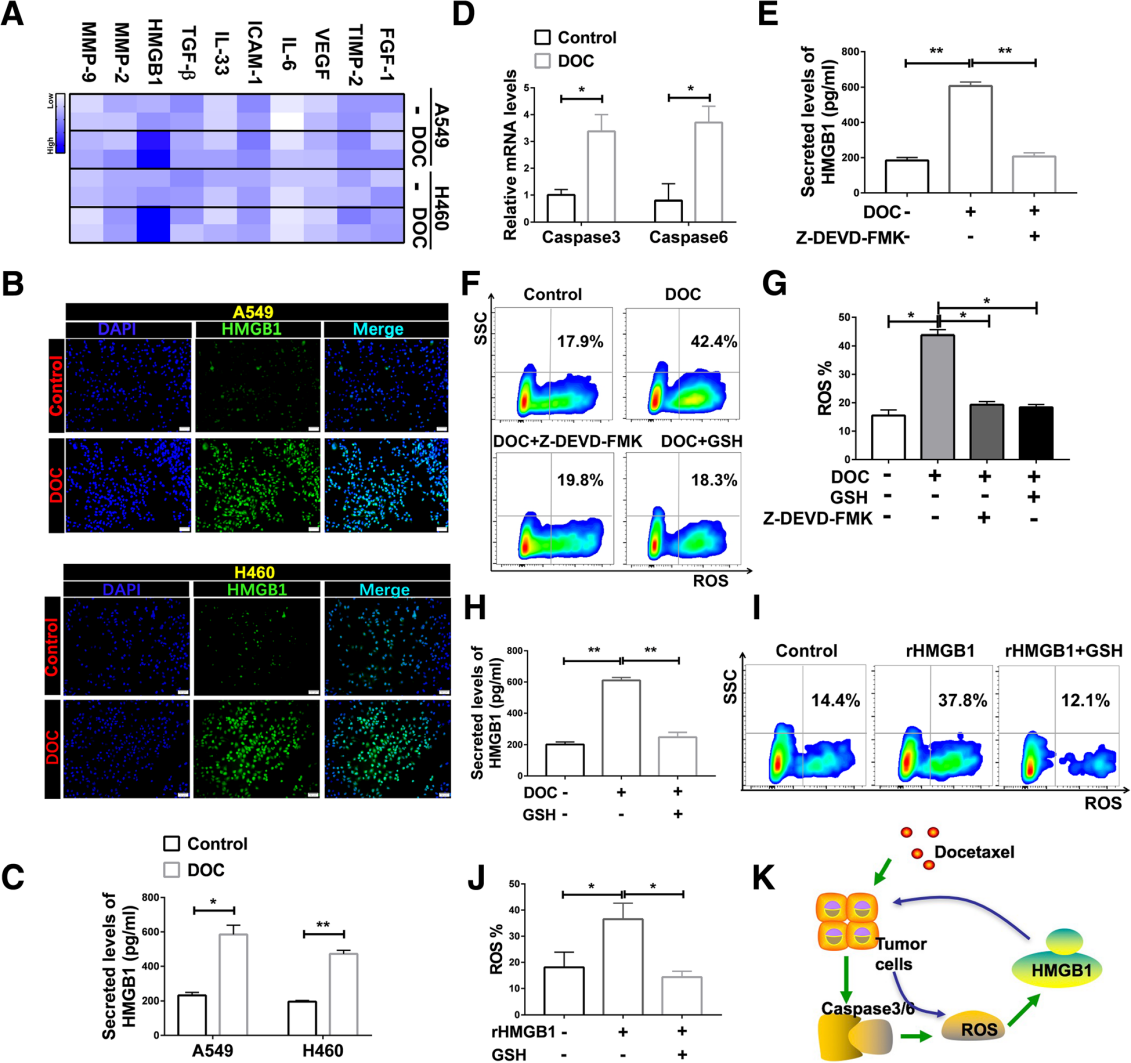
DOC enhanced HMGB1 secretion through a positive feedback process. **a** mRNA expression levels of several cytokines in lung cancer cells were analyzed by qRT-PCR, and are shown as expression heatmap in control group vs DOC treated group. Heatmap color ranging from minimum (white) to maximum (blue) represents the relative gene expression level. **b** HMGB1 protein levels in lung cancer cells in control group vs DOC treated group assessed by immunofluorescence. Scale bars, 50 μm. **c** HMGB1 protein levels in lung cancer cells in control group vs DOC treated group assessed by ELISA. Data are given as means ± SEM. Significant differences between each group are indicated by **p < 0.0001 or *p < 0.05. **d** mRNA expression of caspase 3 and caspase 6 in control and DOC-treated A549 cells. Significance is noted as *p < 0.05. **e** HMGB1 protein levels in A549 cells is upregulated by incubation with DOC, and inhibited by the caspase3/6 inhibitor Z-DEVD-FMK. Significance is noted as **p < 0.0001. **f**, **g** Contents of ROS in A549 cells were evaluated by flow cytometry after treatment with DOC or combined with ROS inhibitor (GSH) or Z-DEVD-FMK, and the differences were significant. **h** The expression of HMGB1 were also analyzed by ELISA after treatment with DOC or combined with GSH. **p < 0.0001. **i**, **j** ROS production in A549 cells treated with recombinant protein HMGB1(rHMGB1) or combined with GSH, as detected by FSC. *p < 0.05. **k** Schematic diagram illustrating the proper release mechanism of HMGB1. The green arrow represents the relationship between DOC and HMGB1, and the blue arrow represents the relationship between HMGB1 and ROS. Data are given as means ± SEM. Significance is noted as **p < 0.0001, *p < 0.05. All of the experiments were independently repeated three times

It has been reported that chemokine release is mainly mediated by cytokines [31, 32]. To understand how DOC stimulated the production of CXCL11, we used qRT-PCR to assess RNA levels of several cytokines in tumor cells after treatment with DOC. HMGB1 was significantly upregulated after stimulation (Fig. 4a). The immunofluorescence and ELISA results verified that DOC induced the upregulation of HMGB1 (Fig. 4b, c). Analysis of tumor cells indicated that DOC strongly induced HMGB1 expression in a dose-dependent manner (Additional file 1: Figure S3A, B).

Analysis of the TCGA database showed that HMGB1 levels were positively related with caspase3 and 6, both of which participate in cell apoptosis (r = 0.2162 and r = 0.1579, respectively; Additional file 1: Figure S3C, D). Thus, we assessed caspase3 and 6 upregulation after treatment with DOC (Fig. 4d). Application of caspase3/6 inhibitor Z-DEVD-FMK reduced the release of HMGB1 in lung cancer cell line A549 (Fig. 4e). It has been reported that the release of HMGB1 is dependent on ROS [33], so we further examined the effect of chemotherapeutic drugs on ROS. DOC induced the release of ROS (from ~ 16.3% to ~ 42.6%), which was inhibited by glutathione (GSH) and Z-DEVD-FMK (reduced to ~ 19.0% and ~ 20.2%, respectively) (Fig. 4f, g). HMGB1, released from the treated tumor cells by DOC, was also inhibited by GSH (Fig. 4h). Interestingly, if treated with recombinant protein HMGB1 (rHMGB1), the tumor cells released more ROS (from ~ 18.1% to ~ 36.5%), ROS released from tumor cells treated with rHMGB1 was also inhibited by GSH (reduced to ~ 14.4%) (Fig. 4i. j). We propose that the release of HMGB1 is a positive feedback process, as illustrated in the schema chart (Fig. 4k).

### HMGB1 promoted the upregulation of CXCL11

**Fig. 5:**
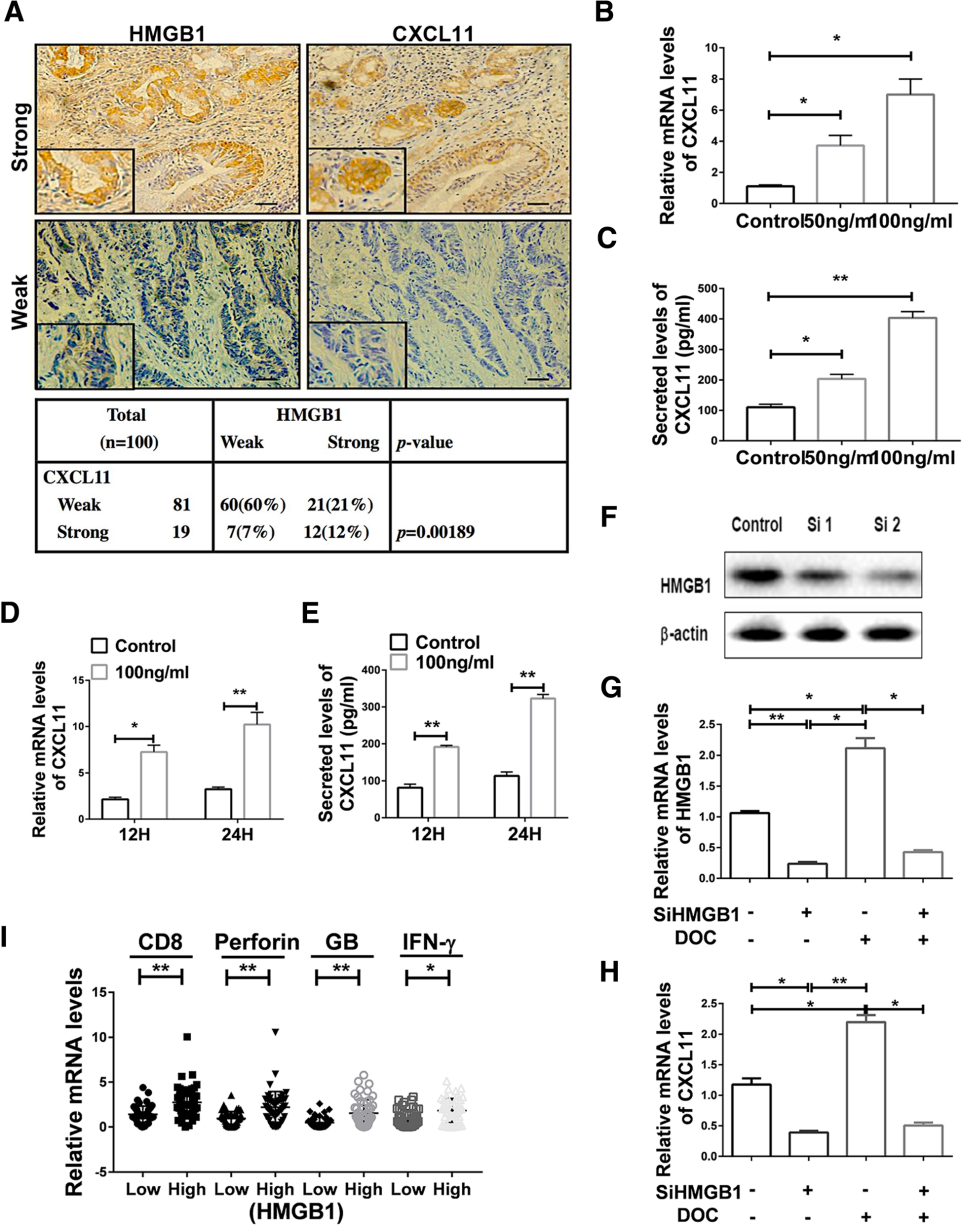
HMGB1 promoted the secretion of CXCL11. **a** Representative immunostaining using anti-HMGB1 and anti-CXCL11 mAbs in serial sections from 2 different patients. Preferential expressions of HMGB1 with CXCL11 in lung cancers (*n* = 100) was analyzed as shown. Figure panel pairs represents images taken with different zooming options; scale bars, 100 μm. **b**, **c** CXCL11 RNA and protein levels in A549 cells after treatment with different doses of rHMGB1 for 24 h, as assayed by qRT-PCR and ELISA. Statistical differences between groups were determined by the Student t test. *P < 0.05; **p < 0.0001. **d**, **e** The release of CXCL11 in A549 cells after treatment with rHMGB1 at different time points was determined by qRT-PCR and ELISA. Statistical differences between groups were determined by the Student t test. *P < 0.05; **P < 0.0001. **f** HMGB1 protein levels in A549 cells after incubation with siRNA. **g**, **h** When HMGB1 transcription was silenced using siRNA, DOC selectively failed to induce HMGB1 and CXCL11 in A549 cells. Data are given as means ± SEM. *P < 0.05; **P < 0.0001. Experiments were independently repeated three times. **i** The relative mRNA levels of CD8, Perforin, Granzyme B and IFN- γ were analyzed in primary tumors secreting high levels (*n* = 50) or low levels of HMGB1 (*n* = 50). Data are given as means ± SEM. *, *P* < 0.05; **, *P* < 0.0001

Serial sections results showed the comparable protein expression tendency of HMGB1 and CXCL11 (Fig. 5a). Further validation showed that rHMGB1 induced expression of CXCL11 in a dose-depended fashion, whereas normal saline had no effect (Fig. 5b, c). CXCL11 expression increased in a time-dependent fashion as well (Fig. 5d). The results were validated at the protein level (Fig. 5e). We detected a significant decrease in the expression of CXCL11 after incubation with glycyrrhizin, an inhibitor of HMGB1, even treated with DOC (Additional file 1: Figure S3E, F). Knockdown HMGB1 was performed to assess the role of DOC inducing CXCL11 (Fig. 5f, g, h, i). And the results showed that HMGB1 was significantly decreased after silencing HMGB1 (Fig. 5f, g and Additional file 1: Figure S3G). Significant downregulation of CXCL11 was also observed in the siHMGB1 group vs control group (Fig. 5h). These data indicate that DOC depends upon HMGB1 to stimulate CXCL11 production. According to the expression levels of HMGB1, the patients were divided into HMGB1 high and low expression groups. NSCLC tissue specimens had greater levels of HMGB1 in cancer patients with increased intratumor expression of CD8, IFN-γ, Granzyme B and Perforin (Fig. 5i). This further verifies that HMGB1 may induce the expression of CXCL11 to enhance the infiltration of CD8^+^ T cells.

### HMGB1 stimulated the release of CXCL11 through the activation of NF-κB signaling

**Fig. 6:**
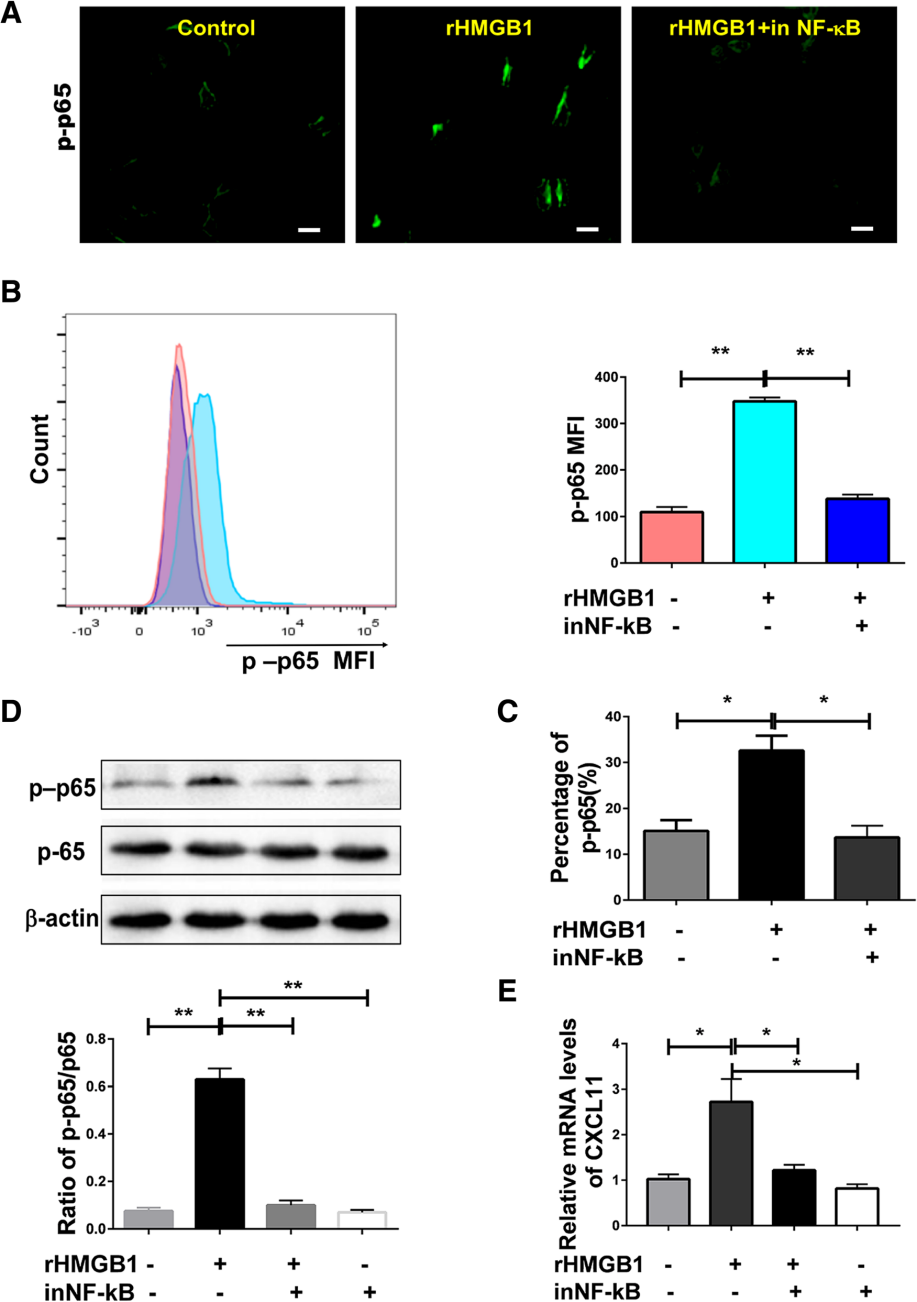
HMGB1 induced release of CXCL11 depends on the activation of NF-κB. **a**, **b**, **c**, **d** Phosphorylation of NF-κB was assessed in A549 cells or cell lysates by immunofluorescence, flow cytometry or western blotting with anti-phosphorylated NF-κΒ antibody as described in methods. The ratio of phosphorylated -NF-κB p65 to NF-κB p65 (p-p65/p65) was calculated. Scale bars, 10 μm. **e** Effects of NF-κB inhibitor on expression of CXCL11 induced by DOC treatment in A549 cells. Data are given as means ± SEM. Significant differences between each group are indicated by **P < 0.0001 or *P < 0.05. Experiments were independently repeated three times and representative results are shown

It has been reported that upregulation of chemokine expression occurs through activation of the NF-κB signaling pathway [34]. Therefore, it is assumed that NF-κB activation was involved in the process of HMGB1-induced CXCL11 expression. As shown in Fig. 6a, HMGB1 stimulated NF-κB phosphorylation, as detected by immunofluorescent staining, and these results were verified by western blot and flow cytometric assay (Fig. 6b, c, d). Additionally, inhibition of NF-kB blocked this effect, as well as HMGB1-induced CXCL11 expression (Fig. 6e). This suggests that NF-κB activation is necessary for HMGB1-induced CXCL11 expression.

### Intratumoural expression of CXCL11 and HMGB1 predicted patients’ survival

CXCL11 and HMGB1 protein levels were assessed in 100 tumor tissues by IHC, and analyzed for associations with clinical outcomes. Higher levels of HMGB1 expression were significantly associated with prolonged overall survival (Additional file 1: Figure S4A, B). Similar results were found with CXCL11 expression (Additional file 1: Figure S4C, D). Thus, upregulation of HMGB1 and CXCL11 might all be the phenotypes of NSCLC with better survival.

### Upregulation of HMGB1 and CXCL11 induced by DOC enhanced the recruitment of HER2-CAR T cells to tumor in vivo

**Fig. 7:**
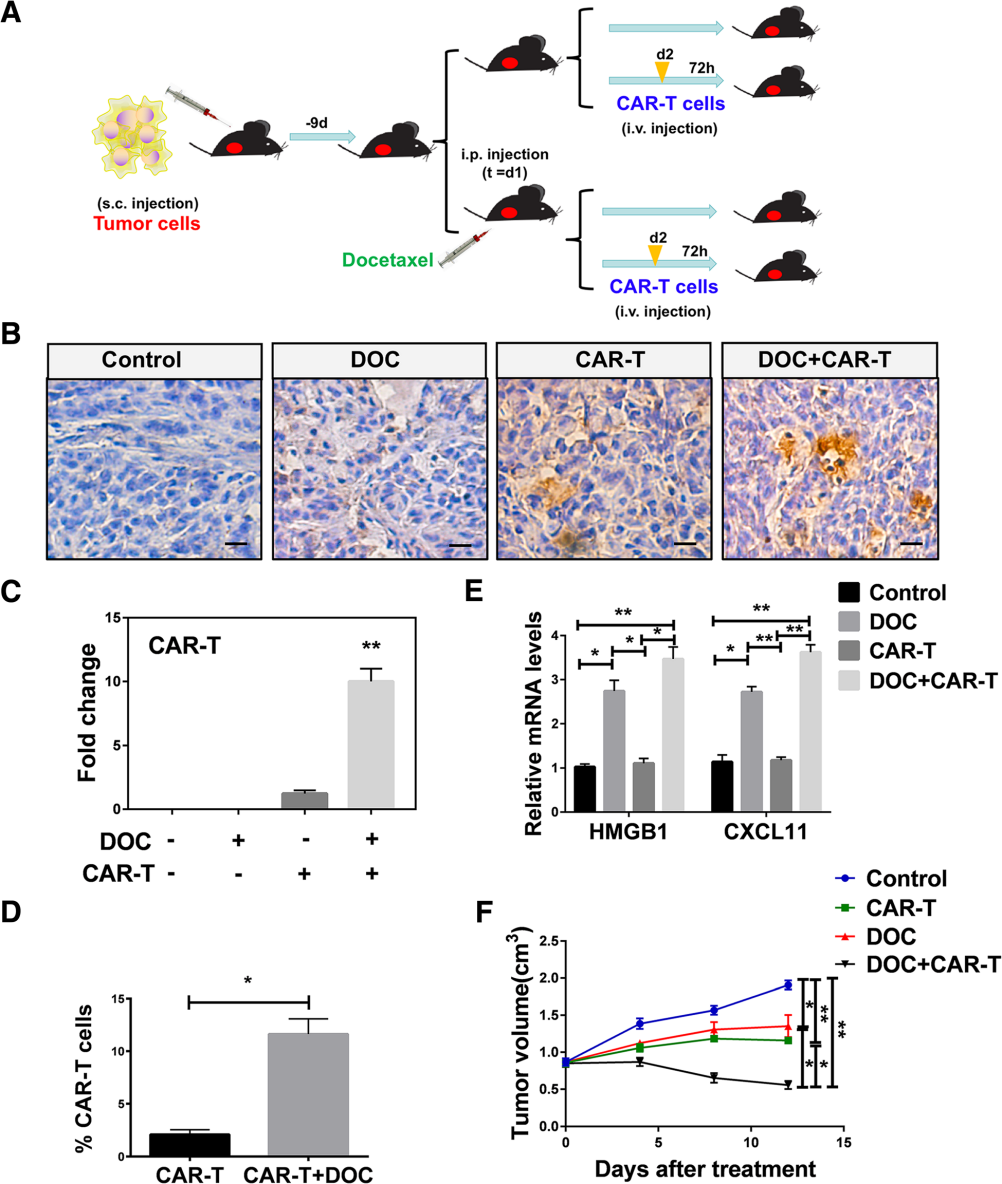
DOC enhanced the recruitment of HER2-CAR T cells to tumor microenvironment in vivo. **a** Treatment scheme showing the timing of mice experiments. **b**, **c** Immunohistochemistry results showed that the expression of HER2-CAR T in untreated, DOC treated, HER2-CAR T cell treated, or DOC in combination with HER2-CAR T treated groups. Scale bars, 20 μm. **d** FSC results showed that the expression of HER2-CAR T in CAR-T cell treated, or DOC in combination with CAR-T cell treated groups. Data are given as means ± SEM. **P < 0.0001. **e** QRT-PCR results showed the expression of HMGB1 and CXCL11 in untreated, DOC treated, HER2-CAR T cell treated, or DOC in combination with HER2-CAR T cell treated groups. An overall difference between the groups was determined by one-way ANOVA. *P < 0.05; **P < 0.0001. **f** Tumor growth was measured in untreated, DOC, CAR-T cell, and DOC in combination with CAR-T cell treated groups. Significant differences between each group are indicated by *, *P* < 0.05; **P < 0.0001

To explore the antitumor effect of DOC in vivo, we established the xenograft mouse model (Fig. 7a). A549 tumor cells which have been demonstrated the presence of HER2 (Additional file 1: Figure S5C) were subcutaneously injected in mice. DOC was administered nine days after injection, with subsequent infusion of HER2-CAR T cells (see HER2-CAR construct and identification of the function of HER2-CAR T cells on Additional file 1: Figure S5). Tumors derived from the DOC in combination with HER2-CAR T cells treated group had the highest infiltration of HER2-CAR T cells compared with those derived from untreated group or a single treated group (Fig. 7b, c, d). QRT-PCR analysis showed that DOC, as a single agent or in combination with HER2-CAR T cells, increased HMGB1 and CXCL11 expression (Fig. 7e). Our results also revealed that DOC alone, HER2-CAR T cells, DOC in combination with infusion of HER2-CAR T cells attenuated the growth of tumor volume (Fig. 7f). DOC in combination with infusion of HER2-CAR T cells attenuated the growth of tumor volume most obviously in comparison with other three groups (Fig. 7f). These results support the hypothesis that DOC treatment inhibits the growth of tumor volume by enhancing HMGB1 and CXCL11 expression, as well as HER2-CAR T cells infiltration, in the xenograft mouse models.

Furthermore, to explore the infiltration of CD8^+^ T cells influenced by DOC in vivo, we injected H460 tumor cells to establish the xenograft mouse model (Additional file 1: Figure S6A). Immunohistochemistry and qRT-PCR analysis showed that DOC, as a single agent or in combination with adoptive transfer of activated CD8^+^ T cells, increased HMGB1 and CXCL11 expression (Additional file 1: Figure S6B, C). Tumors derived from the DOC in combination with CD8^+^ T cells treatment group had the higher infiltration of CD8^+^ T cells compared with that derived from CD8^+^ T cells single treatment group (Additional file 1: Figure S6B, C).

We established a subcutaneous NOD-SCID mouse model (shown as the treatment scheme in Additional file 1: Figure S7A) to identify the effect of CXCL11 and HMGB1 in the chemotherapy-induced recruitment of CD8^+^ T cells into the tumor bed. In this setting, the infiltration of CAR-T cells was found to be upregulated after the i.p. administration of DOC (Additional file 1: Figure S7B). This phenomenon was significantly inhibited by anti-CXCL11 antibody and the inhibitor of HMGB1 and was dependent on the secretion of CXCL11 and HMGB1(Additional file 1: Figure S7B).

## Discussion

Intense infiltration of Tumor Antigen-reactive T cells plays a key role in eradicating tumors. However, these cells often fail to inhibit the tumor because of the reduction of infiltrating effector cells in the tumor microenvironment. Finding a way to increase the infiltration of antitumor immune cells is a pressing matter. Namkoong et al. demonstrated the adjuvant effect of CXCL11-Fc on tumor growth suppression with subsequent increased survival rates in a therapeutic tumor model, which was correlated with enhanced antigen-specific CD8^+^ T cell responses [35]. The roles of CXCR3 ligands, CXCL9, CXCL10 and CXCL11, are well established in terms of their modulatory effects of CD8^+^ T cell migration [36, 37]. We found that CXCL11 was mostly significantly decreased in tumor tissues, and identified nine patients with increased CXCL11 expression in tumor tissues compared to adjacent normal tissue samples (Fig. 2a, b). After carefully looking into the patients’ records, we found that five of the nine patients experienced DOC-based chemotherapy prior to operation, one patient had neoadjuvant radiotherapy before the operation, and the other three cases had no preoperative treatment (Additional file 1: Table S4, *n* = 9). Furthermore, we analyzed all of the 77 lung cancer patients’ characteristics, and found only five patients had experienced DOC-based chemotherapy prior to surgery. We compared the expressions of CXCL10 and CXCL9 between the chemotherapy group (*n* = 5) and the non DOC combined with L-OHP treatment group (*n* = 72), and found that DOC combined with L-OHP treatment did not influence the intratumoural expressions of CXCL10 and CXCL9 (Additional file 1: Figure S2E, F). The levels of CXCL10 and CXCL9 were detected in the two groups treated with (*n* = 3) or without DOC combined with L-OHP (n = 3). These showed similar results to the 77 patients (Additional file 1: Figure S2G, H). We therefore proposed that DOC-based chemotherapy may influence the production of CXCL11, which could possibly reset the tumor microenvironment by regulating CD8^+^ T cells migration [35]. Interestingly, we found that DOC, but not L-OHP, could lead to the release of CXCL11, and we focused on the mechanism that DOC induced the subsequent release of CXCL11 through the induction of HMGB1.

It has been reported that L-OHP also can upregulate the expression of HMGB1 in colorectal cancer cells [38], but our results show that L-OHP neither upregulated the expression of CXCL11 nor stimulated the release of HMGB1 in A549 and H460 cells. In this study, DOC led to the apoptosis of A549 cells and increased the release of HMGB1. These data are inconsistent with the observation that HMGB1 was passively released from human prostate tumors when treated with DOC, while pretreatment with DOC did not lead to HMGB1 secretion or cell death [3]. This discrepancy may be explained by differences in response to dose or differences in the cell lines used. Moreover, HMGB1 levels in the sera of lung cancer patients were increased after DOC combined with L-OHP treatment. This finding is in line with another study that showed increased HMGB1 levels in the serum of cancer patients after chemotherapy [39].

DOC treatment drives HMGB1 release, acting as a potential antitumor immune response inducer in metastatic breast cancer cells [40, 41], but the response of NSCLC patients to neoadjuvant chemotherapy (NCT) was unknown. Our data showed that treatment of A549 or H460 cells with DOC resulted in a significant HMGB1 release in vitro. In in-vivo analysis, HMGB1 levels increased significantly in the DOC treatment group or DOC combined with adoptive transfer of HER2-CAR T cells group. In breast cancer, cytoplasmic HMGB1 expression was associated with TIL levels, but nuclear HMGB1 expression was not associated with TIL levels, and neither cytoplasmic nor nuclear expression of HMGB1 showed prognostic significance in triple negative breast cancer (TNBC) [42]. In our study, we present evidence to support the hypothesis that HMGB1 plays a crucial role in CD8^+^ T cell recruitment. Moreover, we also detected the expression of CD33, CD11b and Foxp3. We found that HMGB1, secreted from tumor cells treated with DOC-based chemotherapy, did not influence the presence of suppressive myeloid (CD33^+^/CD11b^+^ MDSC) or Foxp3^+^Treg cells in tumors (Additional file 1: Figure S8). There are reports that radiotherapy can also produce antitumor benefits in a manner associated with the release of HMGB1, thereby licensing or restoring tumor immunosurveillance capabilities of CD8^+^ T cells against tumor cells [43]. Another study suggests that intracellular HMGB1 is a novel tumor suppressor of pancreatic cancer, adding support to our hypothesis that HMGB1 plays an antitumor role [44]. Overall, our studies establish the idea that HMGB1 plays a crucial role in antitumor immune response by enhancing CD8^+^ T cells accumulation.

An immediate increase in HMGB1 levels correlates with improved outcomes in early breast cancer patients receiving neoadjuvant chemotherapy, and may be a valuable complementary biomarker for early prognosis [45]. Our results indicate that HMGB1 levels correlate with improved outcomes in NSCLC patients. Although there are crucial discoveries revealed by our studies, there is also inconsistence with other reports. In ESCC, HMGB1 is highly expressed, and affects the prognosis of patients by regulating the expression of VEGF-C, promoting lymphangiogenesis and lymph node metastasis [46]. These different tumor models may explain some of the discrepancies in the literature concerning the prognostic value of HMGB1. In our study, we did not compare HMGB1 expression in samples of lung cancer patients and benign tumor patients, making it impossible to assess the application of HMGB1 as a diagnostic marker. However, we suggest that enhancing the HMGB1 might be useful for NSCLC treatment.

## Conclusions

In this study, we have shown a mechanism that DOC can enhance an anti-tumor immune response. Additionally, increased CXCL11 levels measured in tumor tissue of patients treated with DOC suggests that CXCL11 may be involved in eliciting a systemic immune response in patients. Tumor infiltrated CD8^+^ T cells are increased in lung cancer patients after DOC therapy, identifying the activation of apoptosis and the release of HMGB1 and CXCL11 as key events underpinning lung carcinoma cells-elicited leukocyte attraction (Additional file 1: Figure S9). These results support the design of additional clinical studies to measure anti­tumor immune responses after DOC treatment in patients with lung cancer.

## Additional file

Additional file 1: **Figure S1.** Correlation of CD8 and CXCL11 expression in lung cancer tissues. **Figure S2.** DOC had no influence on the release of CXCL10 and CXCL9. **Figure S3.** DOC modulated the release of HMGB1. **Figure S4.** Intratumoural expression of CXCL11 and HMGB1 predicted patients’ survival. **Figure S5.** Anti-tumor activity of HER2-CAR T cells. **Figure S6.** Upregulation of HMGB1 and CXCL11 induced by DOC enhanced the recruitment of CD8+ T cells to tumor microenvironment in vivo. **Figure S7.** Anti-CXCL11 antibody and the inhibitor of HMGB1(Glycyrrhizin) both could reduce the T cell infiltration. **Figure S8.** DOC-based chemotherapy and HMGB1 did not influence the presence of suppressive myeloid (CD33+/CD11b+ MDSC) or Foxp3+Treg cells in tumors. **Figure S9.** Schema chart. **Table S1.** Characteristics of LC patients. **Table S2.** Detailed patient characteristics in additional 6 patients. **Table S3.** Detailed case information of patients with preoperative chemotherapy. **Table S4.** Detailed case information of patients with higher CXCL11 in the tumor tissue. DOCX 5792 kb)

## Abbreviations

DAMP: Damage associated molecular patterns
DOC: Docetaxel
HMGB1: High mobility group box 1
IF: Immunofluorescence
L-OHP: Oxaliplatin
MDSCs: Myeloid-derived suppressor cells
NCT: Neoadjuvant chemotherapy
NSCLC: Non-small cell lung cancer
PBLs: Peripheral blood lymphocytes
PMSF: Phenylmethanesulfonyl fluoride
qRT-PCR: Quantitative real time polymerase chain reaction
rHMGB1: Recombinant protein HMGB1
SiRNA: Short interference RNA
TILs: Tumor-infiltrating lymphocytes
TNBC: Triple negative breast cancer
