# Supplementary material for: Cancer-cell-secreted CXCL11 promoted CD8+ T cells infiltration through docetaxel-induced-release of HMGB1 in NSCLC
Source: J Immunother Cancer. 2019 Feb 11;7:42. doi: 10.1186/s40425-019-0511-6 (PMC6371476; doi:10.1186/s40425-019-0511-6)
Supplement: Supplementary file 1 — Figure S1. Correlation of CD8 and CXCL11 expression in lung cancer tissues. Figure S2. DOC had no influence on the release of CXCL10 and CXCL9. Figure S3. DOC modulated the release of HMGB1. Figure S4. Intratumoural expression of CXCL11 and HMGB1 predicted patients’ survival. Figure S5. Anti-tumor activity of HER2-CAR T cells. Figure S6. Upregulation of HMGB1 and CXCL11 induced by DOC enhanced the recruitment of CD8+ T cells to tumor microenvironment in vivo. Figure S7. Anti-CXCL11 antibody and the inhibitor of HMGB1(Glycyrrhizin) both could reduce the T cell infiltration. Figure S8. DOC-based chemotherapy and HMGB1 did not influence the presence of suppressive myeloid (CD33+/CD11b+ MDSC) or Foxp3+Treg cells in tumors. Figure S9. Schema chart. Table S1. Characteristics of LC patients. Table S2. Detailed patient characteristics in additional 6 patients. Table S3. Detailed case information of patients with preoperative chemotherapy. Table S4. Detailed case information of patients with higher CXCL11 in the tumor tissue. DOCX 5792 kb) [file 40425_2019_511_MOESM1_ESM.docx]

**Supplementary Fig.S1.**

The expression of CD8 and CXCL11 in lung cancer tissues. (A, B) Representative results of CD8, Perforin, Granzyme B and IFN-γ in samples from NSCLC patients by flow cytometry. (C, D) The comparative expression of CXCL11 and CD8 in untreated (without DOC+L-OHP treatment) group (n=3) and chemotherapy (DOC combined with L-OHP) group (n=3). Significance is noted as *p < 0.05. (E) Correlation of CD8 and CXCL11 expression, from the TCGA dataset (<https://xenabrowser.net/datapages/)>. (F) Migration index of CD8^+^ T cells exposed to recombinant CXCL11 with or without CXCR3 antibody, CXCL11 neutralized antibody or CXCR3 antibody. Sup：Supernatant from the cultured tumor tissue. Statistical differences between groups were determined by the Student t test. *p < 0.05. Experiments were independently repeated three times; representative results are shown. (G) Absolute count of CD8^+^ T cells and CD8^-^ cells from TIL exposed to recombinant CXCL11 alone or plus CXCR3 antibody. Statistical differences between groups were determined by the Student t test. (H) Immunohistochemistry (IHC) results of serial sections show that CXCL11 is expressed in tumor cells. Scale bars, 200 μm.


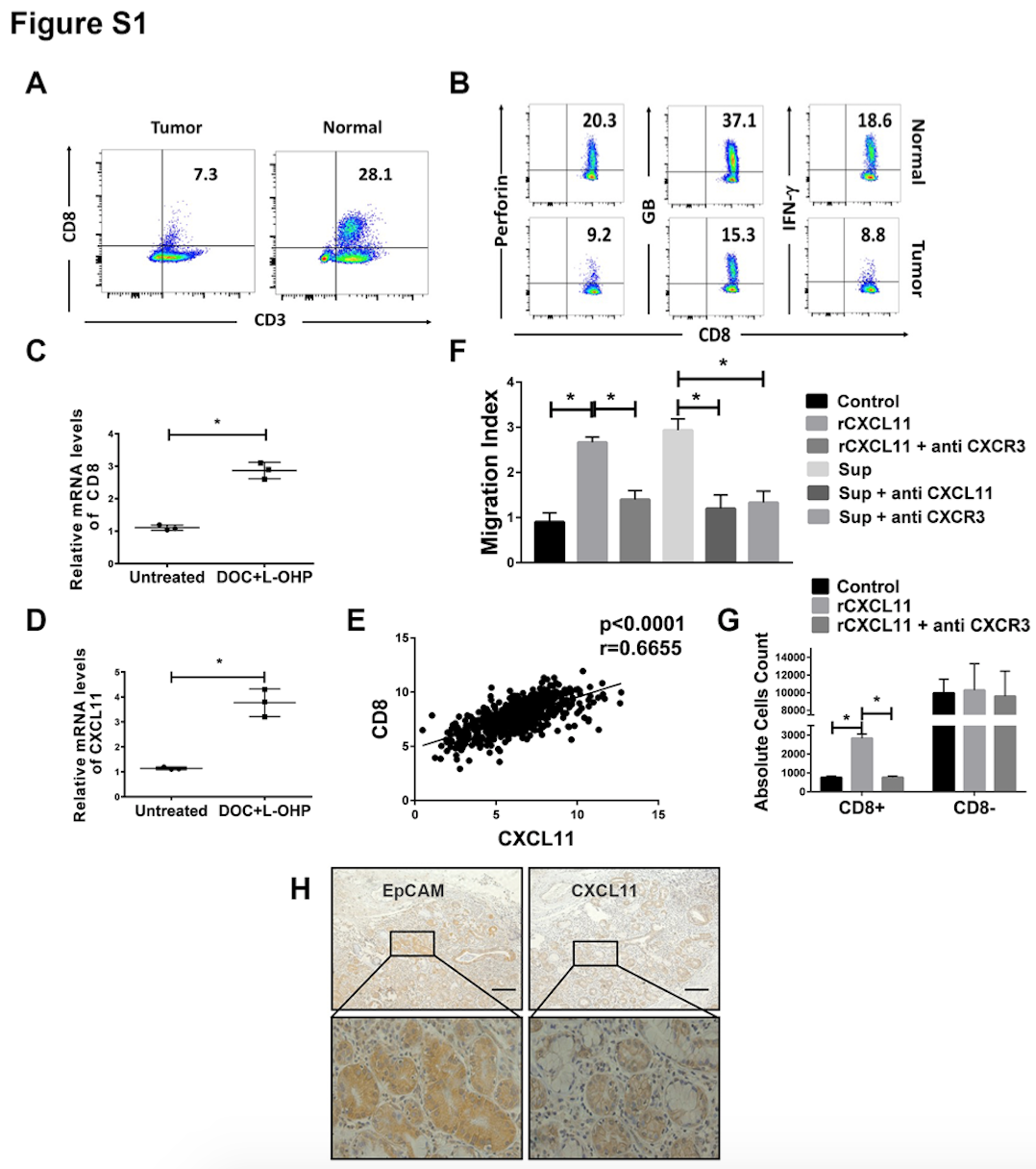


**Supplementary Fig.S2.**

DOC had no influence on the release of CXCL10 and CXCL9. (A-D) Dose and time effects of DOC on RNA expression of CXCL10 and CXCL9 in A549 and H460 lung cancer-derived cells. All the results between each groups showed no significant differences. Experiments were independently repeated three times. (E, F) The comparative expression of CXCL10 and CXCL9 in non DOC combined with L-OHP group (n=72) and DOC combined with L-OHP group (n=5). (G, H) The comparative expression of CXCL10 and CXCL9 in untreated (without DOC+L-OHP treatment) group (n=3) and chemotherapy (DOC combined with L-OHP) group (n=3). Data are given as means ± SEM. NS, non-significant(P>0.05).

**
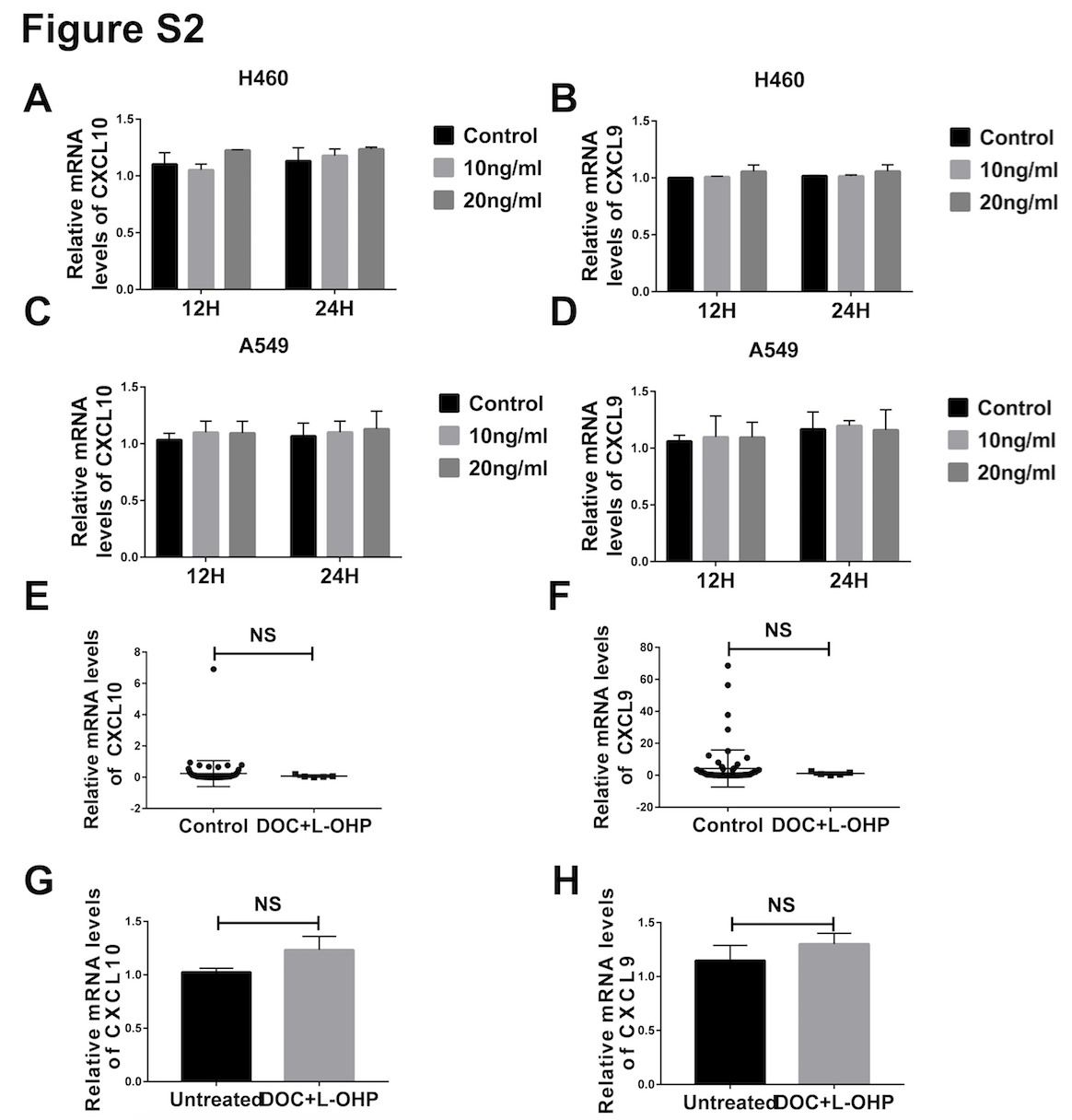
**

**Supplementary Fig. S3.**

DOC modulated the release of HMGB1. (A, B) QRT-PCR detection of HMGB1 expression levels in A549 or H460 cell lines induced by different concentrations of DOC. Significant differences between each group are indicated by **, P < 0.01 or *, P < 0.05. Experiments were independently repeated three times. (C) Correlation of HMGB1 and caspase3 expression from the TCGA dataset (https://xenabrowser.net/datapages/). (D) Correlation of HMGB1 and caspase6 expression from the data of TCGA (https://xenabrowser.net/datapages/). (E, F) Glycyrrhizin abrogated the synergistic effect of CXCL11 expression induced by HMGB1, even if treated with DOC. Significant differences between each group are indicated by *, P < 0.05. Experiments were independently repeated three times. (G) Immunofluorescence showed the downregulation of HMGB1 after using siRNA in A549 cells. Scale bars, 50 μm.

**
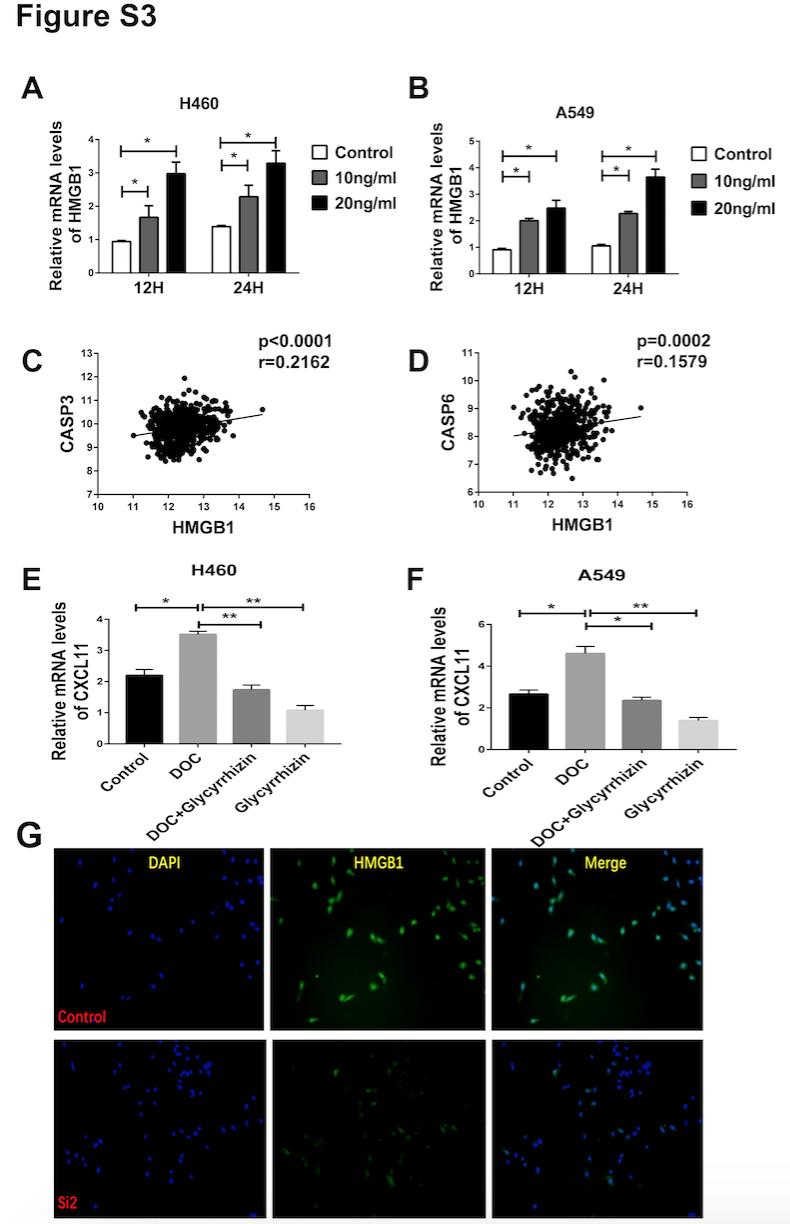
**

**Supplementary Fig. S4**.

Intratumoural expression of CXCL11 and HMGB1 predicted patients’ survival. (A) Representative immunostaining using anti-HMGB1 mAbs in different sections from 2 different patients. Scale bars, 100 μm. (B) Kaplan–Meier survival curve comparing the high and low HMGB1 expression groups. (C) Representative immunostaining using anti-CXCL11 mAbs in different sections from 2 different patients. Scale bars, 100 μm. (D) Kaplan–Meier survival curve comparing the high and low CXCL11 expression groups.


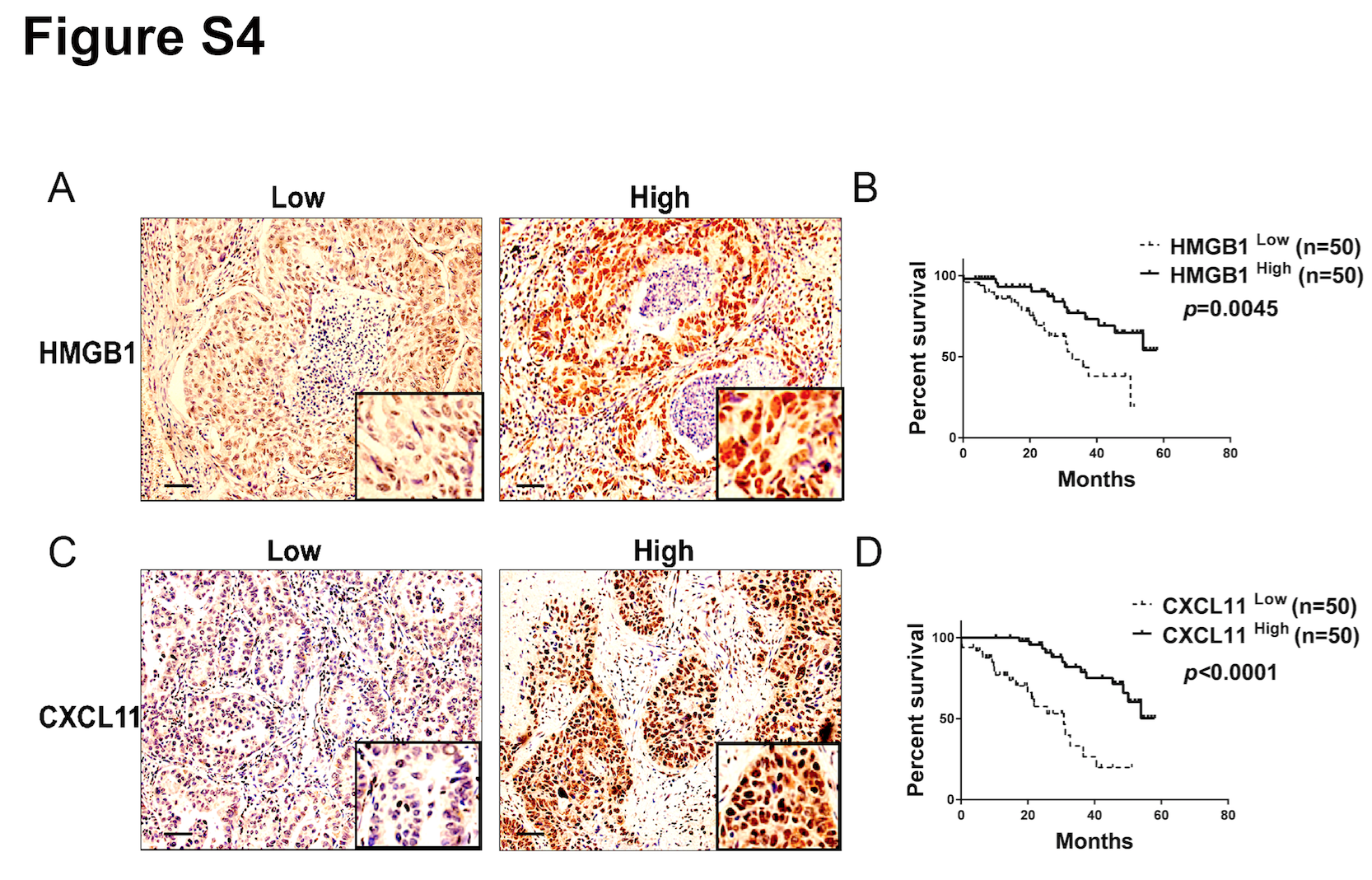


**Supplementary Fig. S5**.

Anti-tumor activity of HER2-CAR T cells. (A) Schematic diagram of the anti-HER2-CAR. The anti-HER2 chimeric antigen receptor construct: we used a 2nd generation anti-HER2 chimeric antigen receptor (HER2-CAR) that features an anti-HER2 scFv, CD8 hinge, CD28 costimulatory domain and CD3-ζ signaling domain. (B) Representative histograms and overall percentage of CAR- expressing transduced T cells. T cells were analyzed by flow cytometry. Activated CD8^+^ T cells were retrovirally transduced with either the anti-HER2 CAR or the empty pMIG II vector as a control. (C) Representative histograms and overall percentage of HER2- expressing tumor cells. (D, E) Representative dot plots and overall percentage of IFN-γ^+^ CD8^+^ T cells in the transduced CAR and pMIG II T cell populations. For in vitro co-culture assays to determine antigen-specific cytokine secretion by the transduced T cells, flow cytometry was performed to determine the amount of IFN-γ secreted following 24 hour co-culture of the transduced T cells with A549 cells. (F, G) The specific lysis of A549 cells co-culture with the transduced T cells were also detected by flow cytometry.


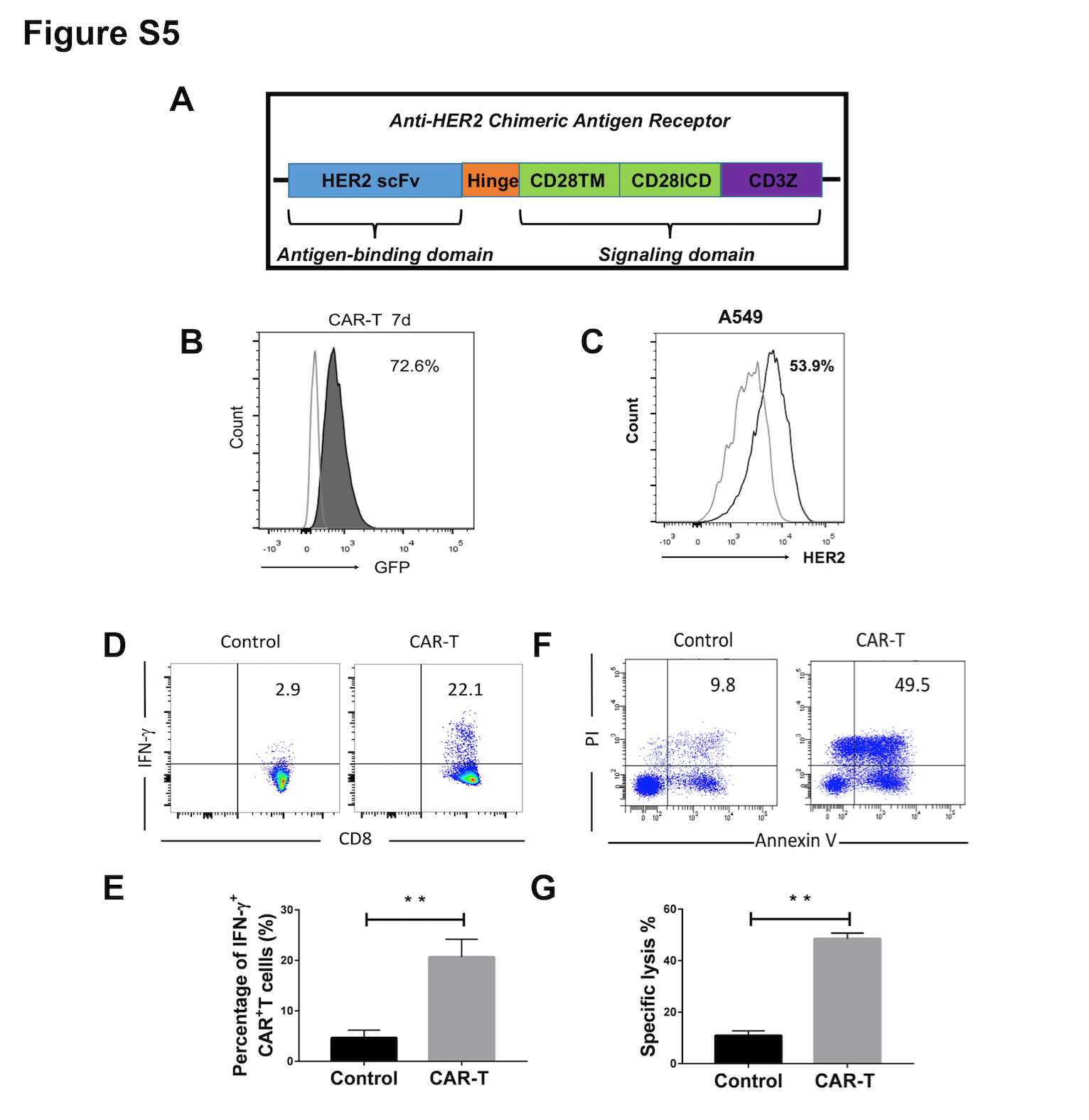


**Supplementary Fig. S6**.

Upregulation of HMGB1 and CXCL11 induced by DOC enhanced the recruitment of CD8^+^ T cells to tumor microenvironment in vivo. Animal studies were approved by the Animal Care and Ethics Committee of the First Affiliated Hospital of Zhengzhou University. Female BALB/c nude mice (5 weeks) were raised in the animal facility of the Experimental Animal Center, Zhengzhou University, under sterile conditions in air-filtered containers. 2 x 10^6^ H460 cells in 100 mL of PBS were injected subcutaneously into nude mice. The nude mice developed tumor nodules approximately 9 days later; they were then randomly divided into 4 groups: untreated, DOC treatment, CD8^+^ T cell treatment, or DOC in combination with CD8^+^ T treatment groups. The PBS-treated group served as the negative control. DOC was injected intraperitoneally on day 10 at a dose of 10 mg/kg. CD8^+^ T cells (1 x 10^7^) were injected intravenously on day 11. After 3 days, animals were sacrificed and the tumors were excised and analyzed by qRT-PCR and immunohistochemistry. (A) Schematic diagram showing the timing of mice experiments. (B) QRT-PCR results showed the expression of HMGB1, CXCL11 and CD8 in untreated, DOC treatment, CD8^+^ T cell treatment, or DOC in combination with CD8^+^ T treatment groups. An overall difference between the groups was determined by one-way ANOVA. *P < 0.05; **P < 0.01. (C) The statistical analysis of immunohistochemistry showed that the expression of HMGB1, CXCL11 and CD8 in untreated, DOC treatment, CD8^+^ T cell treatment, or DOC in combination with CD8^+^ T treatment groups. Significant differences between each group are indicated by *, P < 0.05.

**
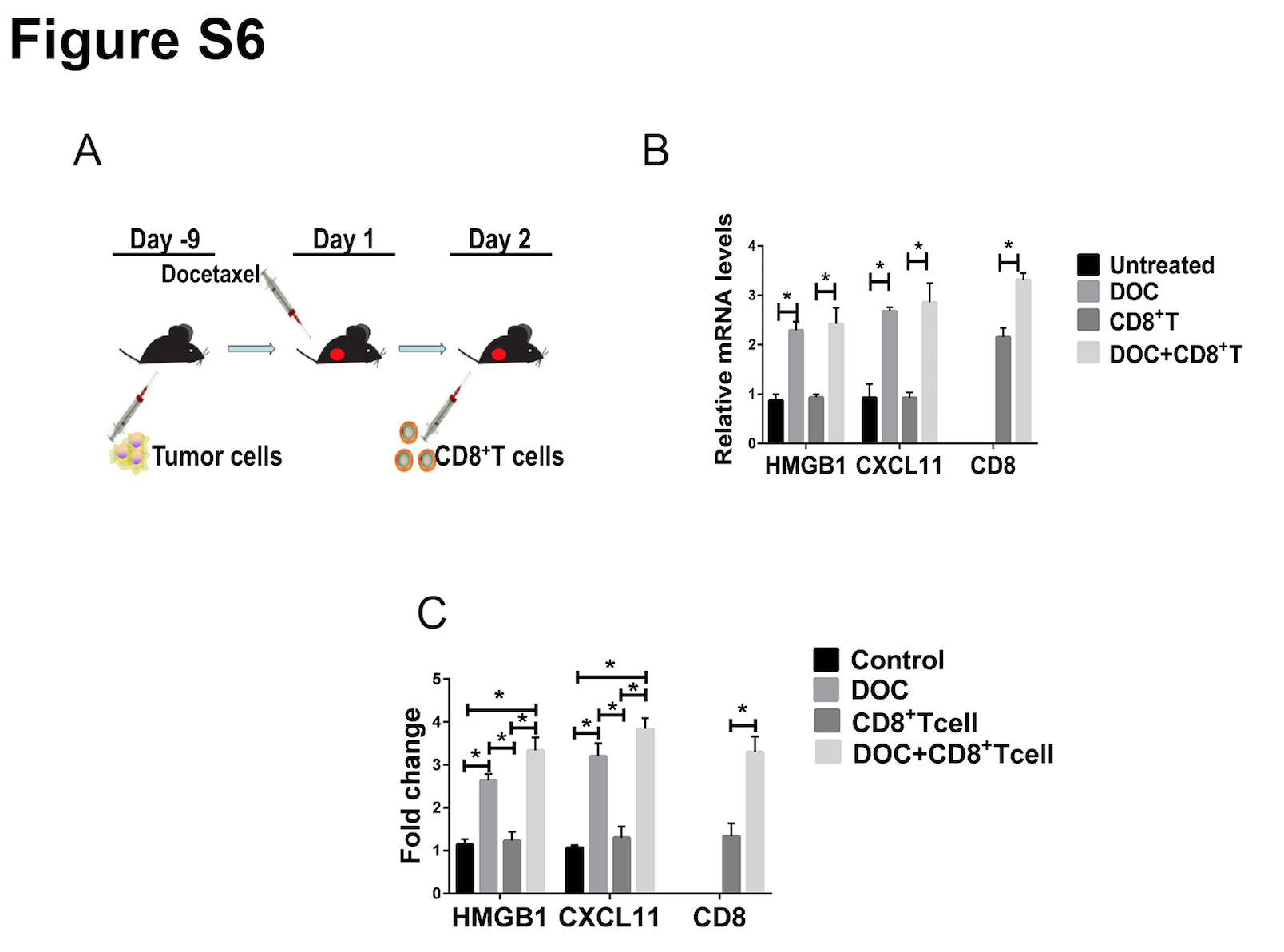
**

**Supplementary Fig. S7**.

Anti-CXCL11 antibody and the inhibitor of HMGB1(Glycyrrhizin) both could reduce the T cell infiltration. Female NOD-SCID mice were raised in the animal facility of the Experimental Animal Center, Zhengzhou University, under sterile conditions in air-filtered containers. The A549 cells (2×10^6^ cells) were subcutaneously injected into the 5-week old female NOD-SCID mice (n=32). The growth of the primary tumors was monitored by external caliper measurement once a week. In approximately 16 days, the mice were blindly randomized into eight groups (shown in the treatment scheme). Half of mice bearing tumors were treated with DOC (10mg/kg, i.p.) and the control group was administered PBS alone. After two hours of treatment, CXCL11 inhibitor (red arrow) or HMGB1 inhibitor (blue arrow) was intratumorally injected. CXCL11 inhibitor (Abcam, ab9955, 1g/kg) or HMGB1 inhibitor (Glycyrrhizin, Tokyo Chemical Industry, 50mg/kg) was administered once every 2 days for 5 days. CAR-T cell (as indicated by yellow arrow) treatment (i.v.) was given on the sixth day. Animals were sacrificed 72 hours post CAR-T cell treatment to analyze the infiltration of CAR-T cells. The tumor size did not exceed 5% of total body weight as permitted in the Institutional Animal Care and Use Committee (IACUC) protocol. (A) Treatment scheme of mice experiments. (B) IHC results showed the expression of HMGB1, CXCL11 and CAR-T in the different treatment groups (n=8). The statistical analysis of IHC showed that the expression of HMGB1, CXCL11 and CAR-T in the 8 groups. Significant differences between each group are indicated by *, P < 0.05. Scale bars, 100 μm.


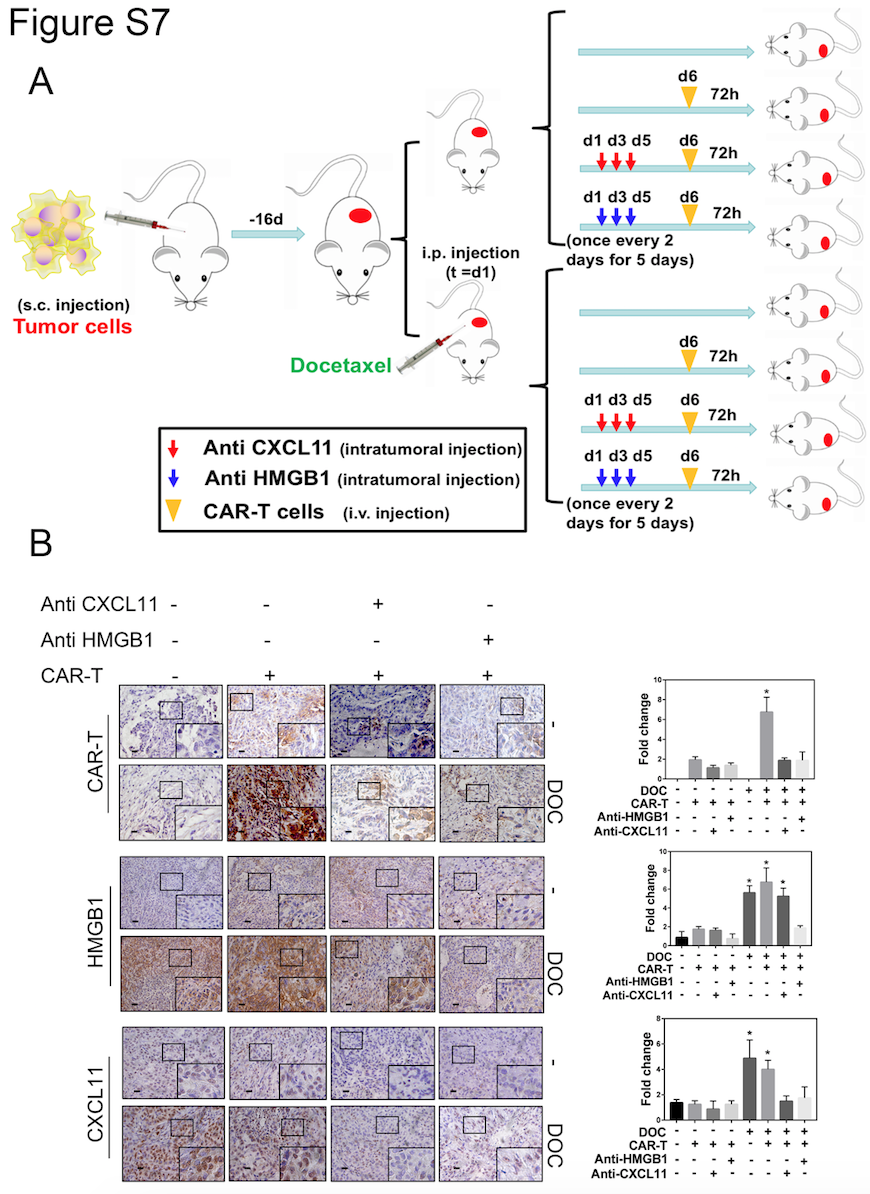


**Supplementary Fig. S8**.

DOC-based chemotherapy and HMGB1 did not influence the presence of suppressive myeloid (CD33^+^/CD11b^+^ MDSC) or Foxp3^+^Treg cells in tumors. (A) The relative mRNA levels of CD33, CD11b and Foxp3 were analyzed in primary tumors with high (n=50) or low levels of HMGB1 (n=50). Data are given as means ± SEM. NS, non-significant (P>0.05). (B-D) The comparative expression of CD33, CD11b and Foxp3 in non DOC combined with L-OHP group (n=92) and DOC combined with L-OHP group (n=8). Data are given as means ± SEM. NS, non-significant(P>0.05).

**
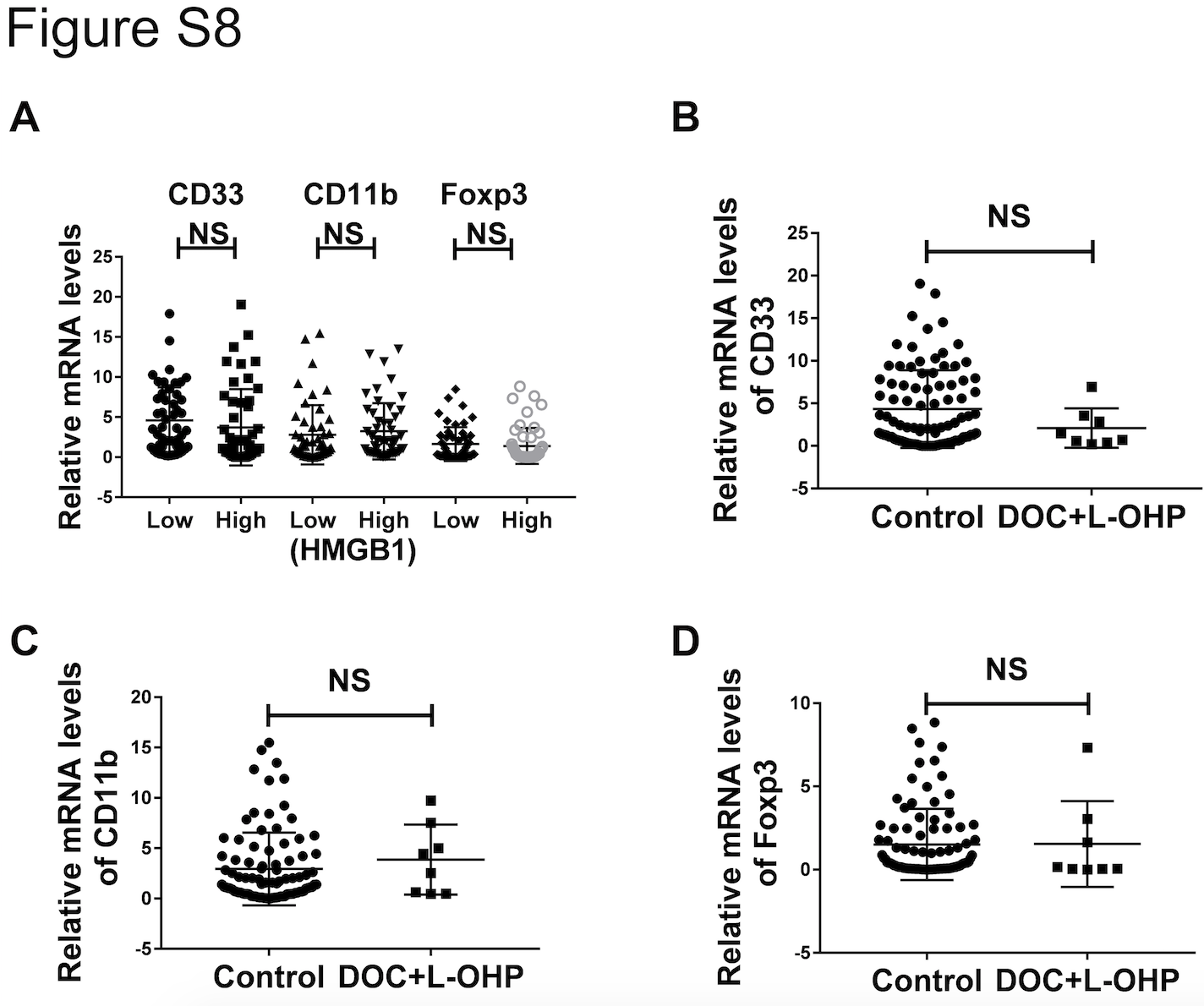
**

**Supplementary Fig. S9**.

Schema chart. DOC treatment significantly increases HMGB1 release in an ROS-dependent manner. Then HMGB1 stimulates the secretion of CXCL11 via NF-κB activation, and CXCL11 subsequently enhances CD8^+^ T cell recruitment to tumor microenvironment. The blue arrow represents the circle about HMGB1 and ROS.


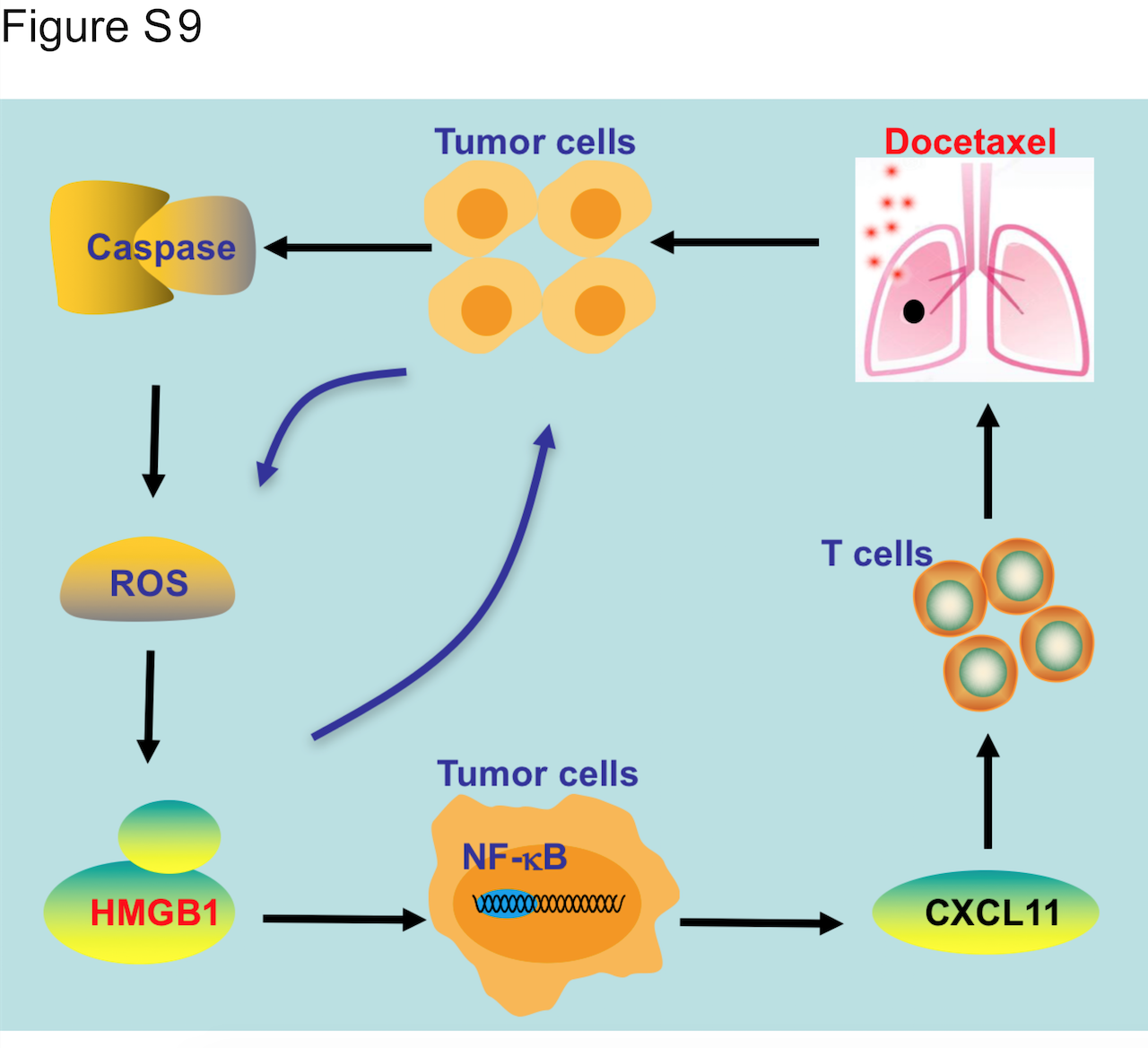


**Table S1 Characteristics of LC patient.**

| Patient characteristics |  |
| --- | --- |
| Total number | 100 |
| Age (year) |  |
| ≤60 | 62(62%) |
| ＞60 | 38(38%) |
| Gender |  |
| Male | 59(59%) |
| Female | 41(41%) |
| UICC stage |  |
| Stages I–II | 78(78%) |
| Stages III–IV | 22(22%) |
| Pathologic grade |  |
| Well | 24(24%) |
| Moderate | 45(45%) |
| Poor | 31(31%) |
| Tumor size |  |
| ≤3cm | 60(60%) |
| ＞3cm | 40(40%) |
| Lymph node metastasis |  |
| None | 72(72%) |
| Present | 28(28%) |
| Brain metastasis |  |
| None | 92(92%) |
| Present  Therapy  Untreated  Docetaxel + Oxaliplatin  Pemetrexed + Cisplatin  Gemcitabine + Cisplatin  Radiotherapy | 8 (8%)  89(89%)  8(8%)  1(1%)  1(1%)  1(1%) |

LC: lung cancer; UICC: Union for international cancer control.

**Table S2 Characteristics of LC patients.**

| **Patient numbers** | **Age (year)** | **Gender** | **UICC stage** | **EGFR**  **or ALK**  **mutation** | | **Pathologic grade** | **Tumor size** | **Lymph node metastasis** | **Brain metastasis** | **Treatment** |  |
| --- | --- | --- | --- | --- | --- | --- | --- | --- | --- | --- | --- |
| **Patient1** | **60** | **Female** | **T1N0M0-Ia** | | **None** | **Moderate** | **＞3cm** | **None** | **None** | **DOC+L-OHP(4cycles)** | |
| **Patient2** | **61** | **Female** | **T1N0M0-Ia** | | **None** | **Moderate** | **＞3cm** | **None** | **None** | **DOC+L-OHP(4cycles)** | |
| **Patient3** | **62** | **Male** | **T1N0M0-Ia** | | **None** | **Moderate** | **＞3cm** | **None** | **None** | **DOC+L-OHP(4cycles)** | |
| **Patient4** | **63** | **Female** | **T1N0M0-Ia** | | **None** | **Moderate** | **＞3cm** | **None** | **None** | **Untreated** | |
| **Patient5**  **Patient6** | **61**  **64** | **Female**  **Male** | **T1N0M0-Ia T1N0M0-Ia** | | **None**  **None** | **Moderate**  **Moderate** | **＞3cm**  **＞3cm** | **None**  **None** | **None**  **None** | **Untreated**  **Untreated** | |

LC: lung cancer; UICC: Union for international cancer control.

**Table S3 Detailed case information of patients with preoperative chemotherapy.**

| **Patient numbers** | **Age (year)** | **Gender** | **UICC**  **stage** | **EGFR**  **or ALK**  **mutation** | | **Pathologic grade** | **Tumor size** | **Lymph node metastasis** | **Brain metastasis** | **Treatment**  **（Before surgery）** |
| --- | --- | --- | --- | --- | --- | --- | --- | --- | --- | --- |
| **Patient1** | **56** | **Male** | **T3N1M0-IIIa** | | **None** | **Poor** | **＞3cm** | **Present** | **None** | **DOC+L-OHP(2cycle)** |
| **Patient2** | **53** | **Male** | **T3N0M0-IIb** | | **None** | **Poor** | **＞3cm** | **None** | **None** | **DOC+L-OHP (2cycles)** |
| **Patient3** | **61** | **Male** | **T3N2M0-IIIa** | | **None** | **Poor** | **＞3cm** | **None** | **None** | **DOC+L-OHP (2cycles)** |
| **Patient4** | **60** | **Male** | **T3N3M0-IIIb** | | **None** | **Moderate** | **＞3cm** | **None** | **None** | **DOC+L-OHP (2cycles)** |
| **Patient5**  **Patient6**  **Patient7**  **Patient8**  **Patient9** | **62**  **66**  **50**  **53**  **60** | **Male**  **Male**  **Male**  **Female**  **Female** | **T4N1M0-IIIa**  **T2N1M0-IIb**  **T4N0M0-IIIa**  **T3N1M0-IIIa**  **T2N2M0-IIIa** | | **None**  **None**  **None**  **None**  **None** | **Moderate**  **Well**  **Poor Moderate**  **Poor** | **＞3cm**  **＞3cm**  **＞3cm**  **＞3cm**  **＞3cm** | **None**  **Present None**  **Present None** | **None**  **None**  **None**  **None**  **None** | **DOC+L-OHP (2cycles)**  **DOC+L-OHP (2cycles)**  **DOC+L-OHP (2cycles)**  **DOC+L-OHP (2cycles)**  **DOC+L-OHP (2cycles)** |

LC: lung cancer; UICC: Union for international cancer control.

**Table S4 Detailed case information of patients with higher CXCL11 in the tumor tissue.**

| **Patient numbers** | **Age (year)** | **Gender** | **UICC**  **stage** | **EGFR**  **or ALK**  **mutation** | | **Pathologic grade** | **Tumor size** | **Lymph node metastasis** | **Brain metastasis** | **Treatment**  **（Before surgery）** |
| --- | --- | --- | --- | --- | --- | --- | --- | --- | --- | --- |
| **Patient1** | **65** | **Female** | **T1N0M0-Ia** | | **None** | **Moderate** | **≤ 3cm** | **None** | **None** | **DOC+L-OHP(1cycle)** |
| **Patient2** | **57** | **Male** | **T2N0M0-Ib** | | **None** | **Moderate** | **＞3cm** | **None** | **None** | **DOC+L-OHP (4cycles)** |
| **Patient3** | **65** | **Male** | **T2N1M0-IIb** | | **None** | **Poor** | **＞3cm** | **Present** | **None** | **DOC+L-OHP (4cycles)** |
| **Patient4** | **62** | **Female** | **T2N2M0-IIIa** | | **None** | **Moderate** | **＞3cm** | **Present** | **None** | **DOC+L-OHP (4cycles)** |
| **Patient5**  **Patient6**  **Patient7**  **Patient8**  **Patient9** | **57**  **56**  **70**  **58**  **59** | **Female**  **Male**  **Male**  **Female**  **Female** | **T2N0M0-Ib**  **T1N0M0-Ia**  **T1N0M0-Ia**  **T1N0M0-Ia**  **T3N1M0-IIIa** | | **None**  **None**  **None**  **None**  **None** | **Moderate**  **Well**  **Well**  **Moderate**  **Moderate** | **＞3cm**  **≤ 3cm**  **≤ 3cm**  **≤ 3cm**  **＞3cm** | **None**  **None**  **None**  **None**  **Present** | **None**  **None**  **None**  **None**  **None** | **DOC+L-OHP (1cycle)**  **Untreated**  **Untreated**  **Untreated**  **Radiotherapy** |

LC: lung cancer; UICC: Union for international cancer control.

**Table S5 The primers used for qRT-PCRs.**

The primers used for qRT-PCR (quantitative real time polymerase chain reaction).

| Gene | Forward primer 5-3 | Reverse primer |
| --- | --- | --- |
| GAPDH | GGAGCCAAAAGGGTCATCACTC | GAGGGGCCATCCACAGTCTTCT |
| CCL4 | AGCTGTGGTATTCCAAACCAAAAGA | ACCTAATACAATAACACGGCACAT |
| CCL19 | GACCCAGAAACCCATCCCTG | TGGTGAACACTACAGCAGGC |
| CCL20 | GCACTCCCAAAGAACTGGGT | AGTTGCTTGCTTCTGATTCGC |
| CXCL9 | GAGTGCAAGGAACCCCAGTA | TTTCTCGCAGGAAGGGCTTG |
| CXCL10 | GAGCCTACAGCAGAGGAACC | GAGAGGTACTCCTTGAATGCCA |
| CXCL11 | GACGCTGTCTTTGCATAGGC | GGATTTAGGCATCGTTGTCCTTT |
| CXCL12 | ATTCTCAACACTCCAAACTGTGC | ACTTTAGCTTCGGGTCAATGC |
| CXCL14 | GGACCCAAGATCCGCTACAG | CTTCGTAGACCCTGCGCTTC |
| CD8 | CCCTGAGCAACTCCATCATGT | GTGGGCTTCGCTGGCA |
| IFN-γ | TCGGTAACTGACTTGAATGTCCA | TCGCTTCCCTGTTTTAGCTGC |
| Granzyme B | CCCTGGGAAAACACTCACACA | GCACAACTCAATGGTACTGTCG |
| Perforin | CAGACAGATGGAAAAGGGAGAT | AGAATGGCGGAGGGCTTAG |
| IL-6 | ATGAGGAGACTTGCCTGGTG | GCATTTGTGGTTGGGTCAG |
| IL-33 | GTGACGGTGTTGATGGTAAGAT | AGCTCCACAGAGTGTTCCTT |
| TGF-β | GCCAGAGTGGTTATCTTTTGATG | AGTGTGTTATCCCTGCTGTCAC |
| HMGB1 | TATGGCAAAAGCGGACAAGG | CTTCGCAACATCACCAATGGA |
| FGF-1 | ACACCGACGGGCTTTTATACG | CCCATTCTTCTTGAGGCCAAC |
| TIMP2 | AAGCGGTCAGTGAGAAGGAAG | GGGGCCGTGTAGATAAACTCTAT |
| VEGF | AGGGCAGAATCATCACGAAGT | AGGGTCTCGATTGGATGGCA |
| ICAM1 | ATGCCCAGACATCTGTGTCC | GGGGTCTCTATGCCCAACAA |
| MMP2 | TACAGGATCATTGGCTACACACC | GGTCACATCGCTCCAGACT |
| MMP9 | TGTACCGCTATGGTTACACTCG | GGCAGGGACAGTTGCTTCT |
| Caspase 3 | AGAACTGGACTGTGGCATTGAG | GCTTGTCGGCATACTGTTTCAG |
| Caspase 6 | TGGTGTCCAACTTCTCTGTCTG | GCTTTGTGTGTGTCTTCCTGAG |
| CD33 | GGCCACTCCAAAAACCTGAC | GACAACCAGGAGAAGATCGGG |
| CD11b | GCCTTGACCTTATGTCATGGG | CCTGTGCTGTAGTCGCACT |
| Foxp3 | ATTCCCAGAGTTCCTCCACAAC | ATTGAGTGTCCGCTGCTTCT |

qRT-PCR: quantitative real time polymerase chain reaction.
